# Extracellular vesicles participate in proteostasis and heat shock adaptation in *Plasmodium falciparum*

**DOI:** 10.1080/22221751.2026.2686468

**Published:** 2026-06-08

**Authors:** Yunuen Avalos-Padilla, Lucía Román-Álamo, Inés Bouzón-Arnáiz, Elsa M. Arce, Diego Muñoz-Torrero, Xavier Fernàndez-Busquets

**Affiliations:** a Barcelona Institute for Global Health (ISGlobal), Hospital Clínic-Universitat de Barcelona, Barcelona, Spain; b Nanomalaria Group, Institute for Bioengineering of Catalonia (IBEC), The Barcelona Institute of Science and Technology, Barcelona, Spain; c Laboratory of Medicinal Chemistry, Faculty of Pharmacy and Food Sciences, University of Barcelona, Barcelona, Spain; d Institute of Biomedicine (IBUB), University of Barcelona, Barcelona, Spain; e Nanoscience and Nanotechnology Institute (IN2UB), University of Barcelona, Barcelona, Spain

**Keywords:** *Plasmodium falciparum*, heat shock, extracellular vesicles, protein aggregation, stress response

## Abstract

Heat shock is a hallmark of clinical malaria, where *Plasmodium falciparum* parasites are exposed to recurrent febrile episodes exceeding 40 °C, which lead to acute proteotoxic stress. Parasite survival under these conditions relies on efficient proteostasis mechanisms and molecular chaperones, yet how stress resilience is coordinated beyond chaperone responses remains poorly understood.

Here, we identify a stress-associated role for extracellular vesicles (EVs) in parasite heat shock adaptation linked to vesicular trafficking mediated by *Pf*Vps60, an Endosomal Sorting Complex Required for Transport (ESCRT) protein. Using a *Pf*Vps60 knockout (*Pf*Vps60KO) line, we show that disruption of ESCRT-dependent vesicular trafficking affects EV cargo composition during thermal stress. Proteomic profiling revealed that 44.8% of EV-associated proteins from *P. falciparum* 3D7 overlapped with a previously defined set of aggregation-prone proteins. Loss of *Pf*Vps60 impaired EV-mediated export of the chaperones *Pf*Hsp70-x and *Pf*Hsp110, altered aggregation dynamics and induced the redistribution of protein aggregates near the parasitophorous vacuole, reduced induction of the cytosolic chaperone *Pf*Hsp70-1, and resulted in early loss of parasite viability following heat shock. Supplementation of *Pf*Vps60KO parasites with EVs derived from heat-stressed 3D7 parasites partially rescued heat shock tolerance in a dose-dependent manner. EVs released shortly after thermal stress were enriched in aggregation-prone proteins and associated with neighbouring uninfected erythrocytes, suggesting EV-mediated intercellular communication during febrile episodes. Together, these findings support a role for EV-associated cargo as a previously unexplored component of *P. falciparum* proteostasis during heat shock adaptation, identifying stress-induced EVs as a potential parasite vulnerability for malaria intervention.

## Introduction

In 2024, malaria remained a major global health burden, with an estimated 270 million cases and 610,000 deaths [1]. The disease is caused by protozoan parasites of the genus *Plasmodium*, among which *Plasmodium falciparum* is the most virulent and the most widespread species in sub-Saharan Africa [2]. The parasite’s life cycle alternates between human and *Anopheles* mosquito hosts [3], exposing it to abrupt thermal fluctuations, ranging from around 25 °C in the insect to 37 °C in the human, and up to 41 °C during febrile episodes [4], a hallmark of clinical malaria which also influences parasite biology. It has been demonstrated that elevated temperatures can enhance cytoadherence of early intraerythrocytic stages [5,6], facilitate synchronization of parasite populations *in vivo* [7], induce stiffening of infected red blood cells (RBCs), contributing to microcirculatory blockages [8], affect proliferation within RBCs [4], and impact on artemisinin susceptibility [9], thereby influencing disease severity. Therefore, understanding how *P. falciparum* withstands and adapts to such thermal fluctuations is essential to unravelling malaria pathogenesis.

In eukaryotic cells, heat stress disrupts protein homeostasis, increasing the risk of proteins to misfold, which potentially leads to the formation of toxic aggregates [10]. To counteract this, cells trigger a response characterized by the transcriptional upregulation of heat shock proteins (Hsp) [11,12]. These molecular chaperones maintain proteostasis by assisting proper protein folding, preventing aggregation, and facilitating degradation of damaged proteins [13,14]. While Hsp expression is primarily induced by elevated temperatures, it can also respond to metabolic and oxidative stress [15,16]. Under physiological conditions, transcription of inducible *hsp* genes remains inactive [17], reflecting the transient, protective nature of this process. In *P. falciparum*, proteostasis under febrile stress relies on a specialized chaperone network, including exported and cytosolic proteins such as *Pf*Hsp70-x and *Pf*Hsp110, which play key roles in protein folding, trafficking and stress tolerance [14,18,19]. However, how these chaperone systems are coordinated with other adaptive mechanisms remains poorly understood.

Beyond the cellular response, stress adaptation can also occur through intercellular communication. The bystander effect describes how naïve cells acquire stress-associated traits after receiving molecular signals from stressed neighbouring cells [20]. This phenomenon often involves the release of extracellular vesicles (EVs) whose production is triggered by adverse conditions, including heat shock [21]. EVs can carry proteins, lipids, and nucleic acids that mediate signalling between cells. Similarly, misfolded or aggregation-prone proteins can be cleared via EVs under proteotoxic stress [22–24], suggesting a role for vesicle-mediated secretion in proteostasis maintenance [25,26].

EVs are heterogeneous in origin and size, being categorized into three groups: apoptotic bodies, microvesicles, and exosomes [27]. The biogenesis of EVs can occur through different pathways, one of which is orchestrated by the sequential recruitment of the Endosomal Sorting Complex Required for Transport (ESCRT), a machinery divided into 4 different complexes (ESCRT-0, -I, -II, and -III) and the accessory Vps4 complex [28]. Although the different ESCRT complexes are well conserved across the eukaryotic lineage, *Plasmodium* lacks most components of ESCRT-0, -I and -II [29], relying instead on a non-canonical ESCRT-III-based pathway. We previously showed that ESCRT-III activation in *P. falciparum* is mediated by the Bro1 domain-containing protein *Pf*Bro1 [30], which binds and induces the recruitment of the Snf7 domain-containing proteins, *Pf*Vps32 and *Pf*Vps60, triggering membrane budding in the model of giant unilamellar vesicles [30,31]. Moreover, deletion of *Pfvps60* significantly reduces EV production [30], demonstrating the importance of this pathway in vesicle biogenesis.

As occurs in other organisms, *P. falciparum*-derived EVs play key roles in parasite-parasite communication, carrying diverse cargoes, such as proteins, mRNA, miRNA, and other small regulatory RNAs [32–34]. These vesicles contribute to essential processes such as RBC remodelling and invasion [35]. However, despite the well-studied participation of EVs during heat shock response in other eukaryotic cells, their role in the malaria parasite under febrile, proteotoxic conditions remains poorly understood. In particular, whether EVs participate in maintaining proteostasis or transmitting stress signals during heat shock in *P. falciparum* has been scarcely explored. In this work, by combining proteomic, imaging, and functional analyses, we investigated the contribution of EV-associated cargoes to stress adaptation and parasite survival. Our results confirm that parasite-derived EVs enriched in aggregative proteins and chaperones are essential for recovery from conditions mimicking febrile stress, suggesting that this mechanism plays a protective role during malaria infection.

## Material and methods

Unless otherwise stated, reagents were sourced from Sigma-Aldrich Corporation (St. Louis, MO, USA).

### P. falciparum cultures

Intraerythrocytic stage *P. falciparum* 3D7 and *Pf*Vps60KO parasites were cultured in 3% hematocrit group B erythrocytes in Roswell Park Memorial Institute (RPMI) medium containing 0.5% Albumax II (Life Technology, Auckland, New Zealand) and 2 mM L-glutamine. Experimental procedures followed previously published protocols [30].

### Heat shock assays

*P. falciparum* cultures of the 3D7 wild type and *Pf*Vps60KO strains were synchronized to ring stages with a 5% sorbitol lysis and grown at 37 °C for 20–25 h, when parasitemia was adjusted to 2%. Cultures were then incubated at 41 °C for 3 h and placed back at 37 °C, following reported protocols [36]. For Western blot assays, samples were collected before heat shock (0 h), at an intermediate point during heat shock (1.5 h), and at 5, 7, and 9 h after the start of the experiment, corresponding to approximately 2, 4 and 6 h after completion of the heat shock pulse. These time points capture mid trophozoite, late trophozoite/early nuclear division, and late trophozoite/schizont stages, respectively. A final time point at 24 h post heat shock was included to analyze longer-term effects on parasite development and progression to the next intraerythrocytic cycle. In parallel, Giemsa-stained thin blood smears were prepared at each time point using standard protocols to analyze parasite morphology and stage distribution. Only well-preserved smears with homogeneous staining were included in the analysis, and parasite stages were assigned based on established morphological criteria. Images were taken with an Olympus IX51 microscope equipped with a cooled CCD Digital Camera (Olympus, Tokyo, Japan).

Parasite survival was quantified by flow cytometry using Syto11 (Thermo Fisher Scientific, Inc., OR, USA) in a 5-laser Cytoflex flow cytometer (Beckman Coulter Life Sciences, CA, USA), with the side- and forward-scatter on a logarithmic scale to determine the cell population, and 10,000 events acquired for each sample. Syto11 fluorescence signal was gated using the B525 channel and survival percentages were calculated as the total parasitemia at the next generation of heat shock-exposed cultures (60–65 h after sorbitol treatment) relative to control cultures and expressed as percentage. This time frame ensured that all parasites had completed the cycle, including those affected by heat shock, which usually showed delayed growth [36]. All experiments were performed in three biological replicates. In all cases, control cultures were included (from 3D7 and *Pf*Vps60KO strains), which were maintained at 37 °C throughout the whole experiment.

### EVs purification

EVs isolation was performed using sequential centrifugation steps followed by size-exclusion chromatography (SEC). This approach has been shown to reduce contamination from soluble proteins compared to other isolation methods. In addition, the combination of ultrafiltration and SEC has been reported to improve EV yield relative to ultracentrifugation-based protocols [37], while SEC itself allows efficient separation of vesicles from free protein components, thereby enhancing sample purity [38]. EV isolation was performed as in [30,39] from conditioned media collected either immediately after the heat shock exposure or following a 24 h recovery period. Parasite cultures were first cleared of cells and debris through sequential low-speed centrifugation steps (400× g, 10 min), followed by two rounds at 2,000× g for 10 min each. The clarified supernatant was concentrated in 100 kDa cut-off AmiconTM Ultra-15 centrifugal filters (Millipore-Merck, Cork, Ireland), and one mL of the concentrated sample was subjected to SEC on Sepharose CL-4B columns equilibrated in 1× phosphate-buffered saline (PBS). Fractions were collected by gravity flow, and EV-enriched fractions were further concentrated to a final volume of 200 µL for downstream analyses.

### EV size and abundance measurements

Particle size distribution and relative particle abundance were analyzed by dynamic light scattering (DLS) as described before with minor modifications [30]. Purified EVs were diluted in 0.22 µm-filtered 1× PBS and gently mixed. Measurements were performed at room temperature using a Zetasizer Nano S instrument (Malvern Instruments, Ltd., Malvern, UK), and each sample was analyzed in triplicate. The hydrodynamic diameter and derived count rate (kilo counts per second, kcps) were recorded to estimate vesicle size and distribution and relative particle concentration.

### Cryogenic transmission electron microscopy (cryoTEM)

For EV imaging, a 1.5 µL aliquot of purified EVs sample was applied onto the carbon side of a glow-discharged Lacey Carbon 300 mesh copper grid (Ted Pella Inc. Redding, CA, USA). Grids were maintained at 100% humidity in a Vitrobot Mark III chamber (FEI Company, Eindhoven, The Netherlands), where excess liquid was automatically blotted with filter paper prior to cryo-immobilization by plunge freezing in liquid ethane. Samples were transferred to a Tecnai Spirit transmission electron microscope (FEI Company, Eindhoven, The Netherlands) using a cryo-holder (Gatan, Pleasanton, USA). Imaging was performed under cryogenic conditions at 120 kV using low-dose settings. Micrographs were acquired with a 1k × 1k CCD Megaview III camera.

### Liquid chromatography-tandem mass spectrometry (LC-MS/MS) protein identification

EV-associated proteins were extracted under denaturing conditions (8 M urea, 50 mM ammonium bicarbonate, pH 8.5) supplemented with protease inhibitors and disrupted by brief sonication. LC-MS/MS analyses were performed using nano-flow liquid chromatography coupled to an Orbitrap mass spectrometer operating in data-dependent acquisition mode. Database searching and peptide identification were carried out using Protein Discoverer with the Sequest HT algorithm and Percolator-based false discovery rate filtering, following workflows as previously described [40]. Search was conducted against a composite database containing the UniProt *P. falciparum* 3D7 proteome, human protein entries, and common laboratory contaminants. Proteins were retained for analysis if detected in at least 2 out of 3 biological replicates for *Pf*Vps60KO samples and at least 3 out of 4 biological replicates for wild type (WT) samples.

Heatmaps were generated using ClustVis (https://biit.cs.ut.ee/clustvis/). Data were log2-transformed (log2(x + 1)), mean-centered and scaled by rows (unit variance scaling). Hierarchical clustering was performed using Euclidean distance and average linkage for both rows and columns.

### Analysis of aggregation-prone proteins from EV-derived extracts

Aggregation tendency was calculated as previously described [41]. Briefly, the TANGO algorithm [42] was run using standard parameters (pH 7.4, 37 °C, ionic strength = 0.25 mM) against the entire *P. falciparum* 3D7 proteome. The resulting β-aggregation propensity (“AGG” value) for each protein was normalized using a min–max scaling approach, where each value was rescaled according to the minimum and maximum AGG values obtained across the full proteome, yielding a normalized scale from 0 (lowest aggregation propensity) to 1 (highest aggregation propensity). These normalized AGG values were the ones assigned to the proteins identified in the EV-derived extracts. Based on commonly used empirical interpretations, values below 0.01 were considered low- or non-aggregative, whereas values above 0.05 were indicative of aggregation-prone proteins [42].

### Protein extraction and Western blot assays

Parasitized RBCs (pRBCs) from the 3D7 or *Pf*Vps60KO strains were washed twice with 1× PBS supplemented with 1× cOmplete^TM^. Parasite pellets were then resuspended in six volumes of 0.15% (w/v) saponin and incubated for 10 min at 4 °C to lyse the host RBC membrane, followed by centrifugation at 10,000× g for 15 min at 4 °C. The resulting pellet was washed thoroughly with 1× PBS containing protease inhibitors and subsequently extracted in RIPA buffer (150 mM NaCl, 2 mM EDTA, 10% glycerol, 1% Triton X-100, 0.5% sodium deoxycholate, 0.2% SDS, 1× cOmplete™, 40 mM tris-HCl, pH 7.4) under continuous agitation at 4 °C. Afterwards, cell lysates were sonicated for 2 s at 40% amplitude and incubated on ice for 15 min to ensure complete protein solubilization. Finally, debris were removed by centrifugation at 20,000× g for 15 min at 4 °C.

For Western blot assays, 20 µg of protein extracts from both parasite lines were resolved by 12% SDS-PAGE. After electrophoresis, proteins were transferred onto nitrocellulose membranes (Cytiva Life Sciences, Marlborough, MA, USA) and blocked overnight at 4 °C with 5% (w/v) skim milk prepared in tris-buffered saline (TBS) containing 0.5% Tween 20 (TBS-T). Membranes were then washed and incubated for 3 h with either rabbit anti-*Pf*Hsp70-1 antibodies (StressMarq Biosciences, Victoria, BC, Canada; 1:10,000) or mouse monoclonal anti-spectrin antibodies (α and β, Sigma-Aldrich; 1:10,000) diluted in TBS-T. Afterwards, membranes were washed 5× with TBS-T and incubated for 1 h with either goat anti-rabbit (Abcam, Cambridge, UK; 1:10,000) or sheep anti-mouse IgG horseradish peroxidase-labelled secondary antibodies (Cytiva Life Sciences), dissolved in TBS-T. Detection was performed using the ECL Prime Western blotting detection reagent (Cytiva Life Sciences). Band intensity was quantified by densitometric analysis using the Fiji software. The relative expression of *Pf*Hsp70-1 in each timepoint was calculated by normalizing the integrated density values to spectrin signal from the same membrane. No apparent changes in spectrin signal were observed across heat shock and control conditions in any of the biological replicates, indicating that spectrin is not affected under the experimental conditions used.

### Confocal fluorescence microscopy

*P. falciparum* parasites from the 3D7 and *Pf*Vps60KO strains were seeded in an 8-well µ-slide chamber (ibidi GmbH, Germany). Briefly, pRBCs were washed twice with warm 1× PBS and deposited into wells pre-coated with 5 mg/mL concanavalin A. Cells were incubated for 10 min at 37 °C to allow adhesion, after which unbound RBCs were gently removed by washing with 1× PBS at 37 °C. For fixed preparations, adhered pRBCs were incubated with 4% paraformaldehyde for 20 min at 37 °C and thoroughly washed with 1× PBS. Live or fixed cells were then incubated for 30 min at 37 °C with 0.5 µM YAT2150 and 5 µg/mL Hoechst 33342. After staining, samples were rinsed with 1× PBS to remove excess dyes. Fluorescence imaging was performed using a Leica TCS SP5 confocal microscope (Leica Microsystems GbmH, Germany) equipped with a 63×/1.4 NA oil immersion objective. Hoechst 33342 was excited with a 405 nm diode laser, and YAT2150 with a 488 nm diode-pumped solid-state laser. Emission was collected between 415–460 nm for Hoechst 33342 and 490–590 nm for YAT2150.

### Quantitative analysis of protein aggregation levels in live P. falciparum cultures

Aggregation tendency was calculated as previously described [43]. For the quantitative analysis of protein aggregation, *P. falciparum* 3D7 and *Pf*Vps60KO parasites were synchronized to the trophozoite stage (20–25 h post infection, hpi) via sorbitol lysis. The resulting cultures were adjusted to 3% hematocrit and 2% parasitemia in complete RPMI medium and one sample from each line was subjected to heat shock at 41 °C for 2 h in a water bath. This shorter exposure was selected to minimize excessive parasite death during EV supplementation, thereby allowing the determination of functional rescue during the recovery phase. Corresponding control samples were maintained at 37 °C under identical conditions.

Immediately after this treatment (t0), 5 mL of each culture sample were harvested by centrifugation (1,500× g, 5 min), and the resulting pellet was resuspended in 300 µL of osmotic lysis buffer (4.5 mg/mL NaCl, supplemented with 1× cOmplete^TM^ EDTA-free protease inhibitor). An additional 5-mL aliquot from each condition (37 °C and heat shock) was collected after further incubation at 37 °C for 4 h.

All samples were incubated overnight at 4 °C under constant stirring to ensure complete lysis. Lysates were clarified at 14,000× g for 5 min, and protein content was determined using the Pierce BCA Protein Assay Kit (Thermo Fisher Scientific, Waltham, MA, USA). For the aggregation assay, 30 µg of total protein per sample were adjusted to 100 µL with 1× PBS and loaded in triplicate into a black 96-well plate (Greiner Bio-One, Madrid, Spain). Thioflavin T (ThT) was added to a final concentration of 25 µM from a freshly prepared stock in 1× PBS. Plates were incubated for 15 min at room temperature in the dark under gentle stirring. Fluorescence emission spectra (500–700 nm) were acquired using an excitation wavelength of 450 nm in an Infinite® M Plex multimode microplate reader (Tecan Trading AG, Männedorf, Switzerland). Fluorescence values were corrected by subtracting a ThT blank prepared in 1× PBS under identical conditions. Data was analyzed by calculating the area under the curve (AUC) from each emission spectrum using GraphPad Prism software (v10.4.2). Statistical analysis was performed using a two-way ANOVA to evaluate the effects of strain and treatment at each time point.

### Extracellular vesicle supplementation during heat shock survival assays

For the heat shock survival assays, parasites from the 3D7 and *Pf*Vps60KO lines were synchronized to the trophozoite stage (24–28 hpi) using a 70% Percoll gradient followed by sorbitol lysis. Synchronized cultures were adjusted to 3% hematocrit and 2% parasitemia and divided into 1 mL aliquots in complete RPMI. Heat shock was performed by incubating parasite aliquots in a water bath at 41 °C for 2 h, while control samples were kept at 37 °C under identical conditions.

For EV supplementation experiments, *Pf*Vps60KO parasites were incubated during heat shock in the presence of four different EV preparations derived from 3D7-parasitized erythrocytes: three obtained from heat shocked cultures (s1, s2, and s3) and one obtained from a culture maintained at 37 °C (s4). All EV preparations were added at a fixed volume (25 µL per mL of culture, corresponding to 2.5% of total volume), but containing decreasing EV concentrations: s1 (7437.7 ± 47.9 kcps/mL), s4 (4153.0 ± 36.4 kcps/mL), s2 (2781.0 ± 51.2 kcps/mL) and s3 (1017.0 ± 28.9 kcps/mL). Additionally, a 1× PBS control (2.5% v/v) was included. After heat exposure, cultures were returned to standard culturing conditions and allowed to recover for 24 h. Parasite survival was quantified by flow cytometry using Syto11 (Thermo Fisher Scientific, Inc.) in a 5-laser Cytoflex flow cytometer (Beckman Coulter Life Sciences, CA, USA), as previously described.

### Statistical analyses

Statistical analyses were performed using GraphPad Prism (version 10.4.2, GraphPad Software). Data are presented as mean ± standard error of the mean (SEM) unless otherwise indicated. The specific statistical tests applied for each experiment are described in the corresponding figure legends. For comparisons involving more than two groups, one-way or two-way analysis of variance (ANOVA) was used as appropriate, followed by either Tukey’s or Šídák’s multiple comparison tests to analyze pairwise differences. For comparisons between two groups, unpaired Student’s t-test was applied. Statistical significance was defined as *p* < 0.05.

## Results

### The disruption of PfVps60 in P. falciparum leads to an impairment in the export of the heat shock proteins PfHsp70-x and PfHsp110

**Figure 1.**
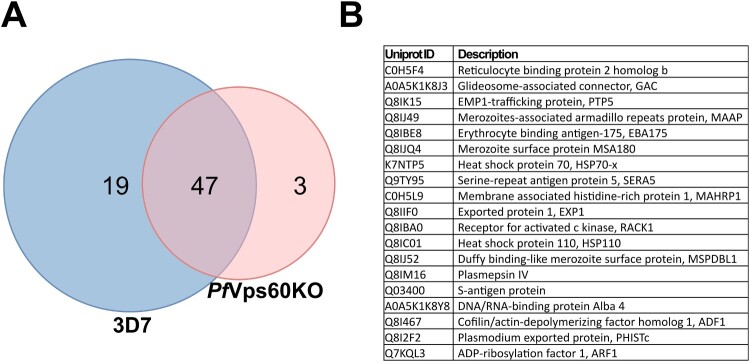
Comparative proteomic analysis of EVs from *P. falciparum* 3D7 and *Pf*Vps60KO strains. (A) Venn diagram showing the distribution of proteins identified in EVs derived from the WT 3D7 strain and from the *Pf*Vps60KO mutant, with the overlapping region indicating proteins shared between both samples. (B) List of the 19 proteins found in 3D7 EVs and absent in EVs derived from the *Pf*Vps60KO strain.

Given the central role of molecular chaperones in parasite proteostasis under stress conditions, we first investigated whether disruption of the ESCRT-III protein *Pf*Vps60 affects the EV-associated export of stress-related proteins in *P. falciparum.* According to previous findings from our group, knockout (KO) of the *Pfvps60* gene resulted in a significant reduction in EVs production compared to the wild type (WT) 3D7 strain, and impaired export of other ESCRT subunits, including *Pf*Vps32 and *Pf*Bro1, to the host cell cytoplasm [30]. Despite the reduced EV yield, cryoTEM analysis of *Pf*Vps60 preparations confirmed the presence of nanosized vesicular structures with morphology consistent with EVs, including heterogeneous populations with occasional electron-dense vesicular material (Figure S1). These observations indicate that *Pf*Vps60 disruption does not abolish vesicle formation but rather affects EV abundance and/or cargo composition. WT 3D7-derived EV morphology was previously characterized in our group [39]. To further investigate whether *Pf*Vps60 loss broadly affects vesicular trafficking of proteins relevant to proteostasis, we performed a comparative LC-MS/MS proteomic analysis of EVs isolated from WT and *Pf*Vps60KO pRBCs. A total of 111 *P. falciparum* proteins were initially identified in four WT and three *Pf*Vps60KO biological replicates. After excluding proteins not consistently detected across biological replicates, 69 were retained for analysis (Table S1, see Material and Methods). A heatmap representation of protein abundance across samples revealed distinct clustering patterns between WT- and *Pf*Vps60KO-derived EVs (Figure S2). Among the analyzed proteins, 19 were absent in EVs from the *Pf*Vps60KO strain (Figure 1A). In particular, several of the missing proteins have been previously reported in EVs isolated from clinical samples, including MSA180, GAC, SERA5, MAHRP1, Alba 4, EXP1 and EBA175 [35]. Additionally, the export of EV-associated proteins such as RACK1, ADF1, PHISTc and ARF1, previously characterized in pRBC cultures [32,33], was also disrupted in the *Pf*Vps60KO strain. Interestingly, EV-associated export of the molecular chaperones *Pf*Hsp70-x (K7NTP5) and *Pf*Hsp110 (Q8IC01) was not found in the KO line (Figure 1B).

Together, these results indicate that the ESCRT-III machinery is required to maintain the protein composition of EVs released by infected erythrocytes. Given the selective loss of EV-associated chaperones observed in the *Pf*Vps60KO line, we next examined whether disruption of ESCRT-mediated vesicular trafficking compromised the parasite’s ability to adapt to febrile heat stress.

### Heat shock induces a protective response in the P. falciparum 3D7 WT line but not in the PfVps60-deficient strain

During the blood stage of malaria infection, recurrent febrile episodes expose *P. falciparum* to periodic thermal stress. As in other eukaryotic cells, these temperature fluctuations promote protein unfolding and aggregation, thereby activating protective chaperone-mediated mechanisms [19,44]. Among the most studied parasite molecular chaperones, *Pf*Hsp70-1 (Q8IB24) is a cytosolic protein essential for parasite survival that is upregulated in response to stress [45]. Other chaperones, including *Pf*Hsp90 and a large family of exported Hsp40 co-chaperones, cooperate with *Pf*Hsp70-1 to maintain proteostasis under fluctuating host temperatures [18,46]. *Pf*Hsp70-x, a dispensable exported chaperone unique to *P. falciparum* [47], and *Pf*Hsp110, which prevents protein aggregation *in vitro* and protects the parasite’s asparagine-rich proteome during febrile episodes [19], are also part of this network.

**Figure 2.**
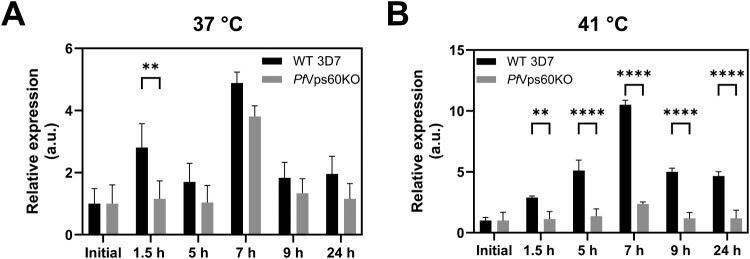
*Pf*Hsp70-1 expression in *P. falciparum* WT and *Pf*Vps60KO lines under heat shock stress. Protein expression levels of *Pf*Hsp70-1 in parasites synchronized at the trophozoite stage (initial), either (A) maintained at 37 °C or (B) subjected for 3 h to heat shock at 41 °C, followed by recovery at 37 °C. The indicated time points represent hours elapsed since the start of the heat shock treatment. Protein levels were normalized to spectrin. Quantification was obtained by densitometric analysis of Western blot signals (shown in Figure S3 and Supplementary S1 Data). Data represents the mean ± SEM of three independent experiments. a.u., arbitrary units. Statistical analysis was performed using a two-way ANOVA comparing each condition to the corresponding 3D7 control. **p ≤ 0.01, ****p ≤ 0.0001.

To analyze whether *Pf*Vps60 disruption impacted the response of *P. falciparum* to heat shock, trophozoite stage parasites were incubated for 3 h at 41 °C, and samples were collected before, during, and after heat shock exposure. Subsequently, expression levels of *Pf*Hsp70-1 (whose export is not affected in the KO strain, see Table S1) were analyzed by Western blot and compared with control parasites maintained at 37 °C (Figure S3). Under normal conditions, mostly non-significant differences in *Pf*Hps70-1 expression were detected between the WT 3D7 and *Pf*Vps60KO lines (Figure 2A). However, during heat shock treatment, *Pf*Hsp70-1 expression in the KO strain failed to increase following thermal stress, in contrast to the robust induction observed in WT parasites (Figure 2B). It is important to point out that the peak of expression detected at 7 h matches with schizont formation, a stage likely requiring enhanced chaperone activity to support cell division.

**Figure 3.**
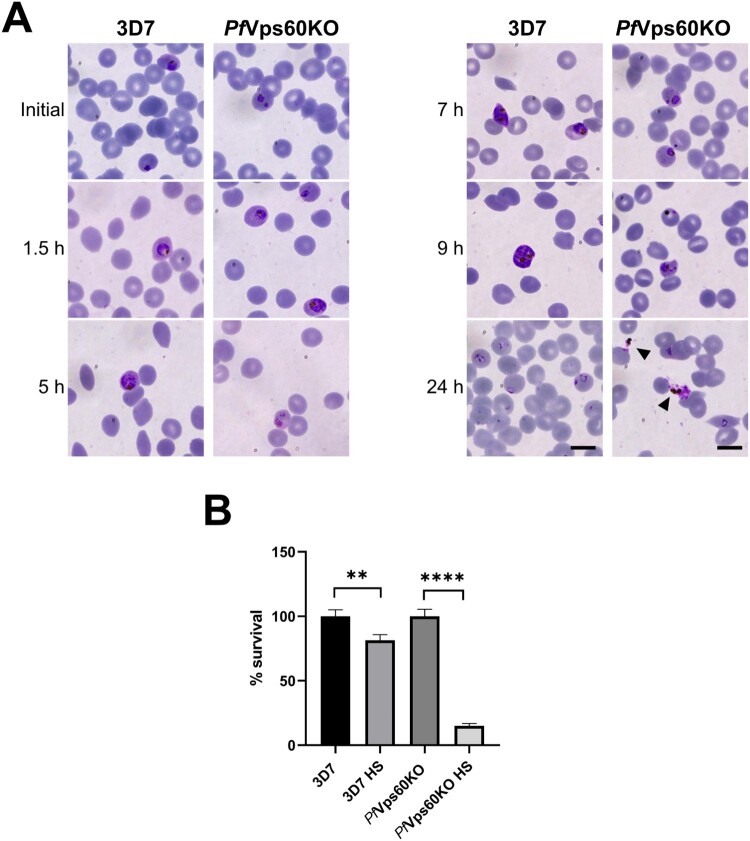
Heat shock impairs recovery of *Pf*Vps60KO parasites. (A) Representative Giemsa-stained images of *P. falciparum* cultures at the indicated times after heat shock exposure (41 °C, 3 h) of synchronized trophozoite stage parasites from the 3D7 or *Pf*Vps60KO lines. Arrowheads indicate ruptured pRBCs. Scale bar = 10 µm. (B) Parasite survival following heat shock quantified 24 h post-treatment for 3D7 and *Pf*Vps60KO lines, expressed as parasitemia determined by flow cytometry using Syto11 staining. Data represent mean ± SD of 3 independent biological replicates. Statistical analysis was performed using a Student’s *t*-test comparing each condition to the corresponding untreated control. **p ≤ 0.01, ****p ≤ 0.0001.

Giemsa-stained preparations revealed that, 24 h after heat shock treatment, WT parasites progressed through the intraerythrocytic cycle and reached ring stage at the expected time (Figure 3A). In contrast, *Pf*Vps60KO parasites exposed to the same conditions exhibited a marked loss of fitness, evidenced by the presence of ruptured cells and pyknotic nuclei (Figure 3, arrowheads). To quantitatively measure parasite recovery following heat shock, survival was independently evaluated by flow cytometry using Syto11 staining. Consistent with morphological observations, flow cytometry analysis revealed that approximately 80% of WT parasites survived heat shock, whereas survival of *Pf*Vps60KO parasites dropped to ∼15% 24 h post-treatment (Figure 3B). Together, these results indicated that *Pf*Vps60-deficient parasites fail to efficiently recover from febrile stress, leading to loss of viability and impaired progression through the intraerythrocytic cycle. It is important to point out that flow cytometry does not distinguish between actively replicating and dormant parasites, which may retain viability while exhibiting reduced metabolic activity. Therefore, we cannot exclude that a fraction of the surviving population corresponds to transiently growth-arrested parasites.

### Heat shock triggers a rapid but transient increase in EV production

**Figure 4.**
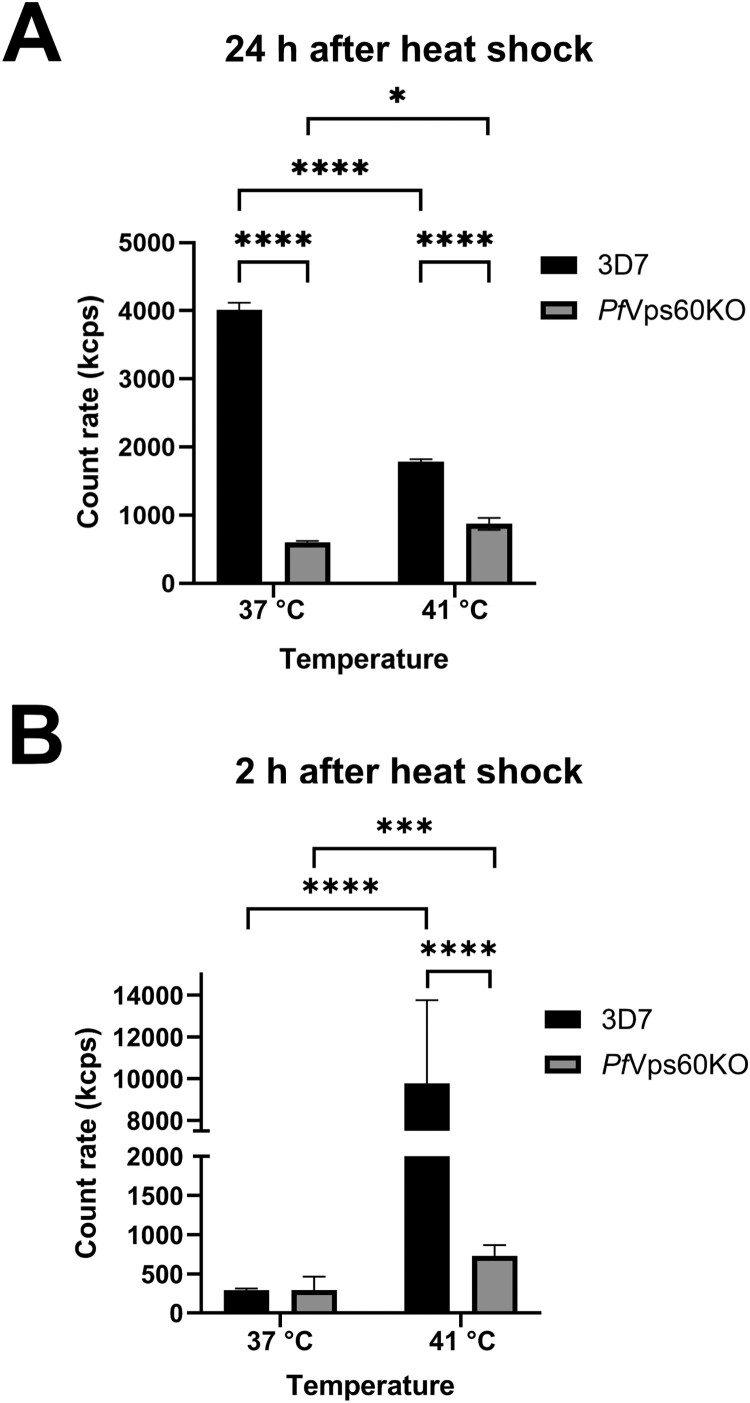
Relative EV abundance in *P. falciparum* 3D7 and *Pf*Vps60KO lines after heat shock. Derived count rate of purified EVs from three independent replicates, expressed in kilo counts per second (kcps), measured at (A) 24 h and (B) 2 h post-heat shock treatment. Data represent mean ± SD of 3 independent biological replicates. Statistical analysis was performed using one-way ANOVA followed by Tukey’s multiple comparisons test comparing each condition to the corresponding control. *p ≤ 0.05, ***p ≤ 0.001, ****p ≤ 0.0001.

To further explore the reduced survival observed in the *Pf*Vps60KO line after heat shock, we examined EV production under conditions of thermal stress. Synchronized parasites from the WT and KO lines were exposed to heat shock (41 °C, 3 h) and subsequently returned to 37 °C; a control sample was maintained at 37 °C throughout the assay. When EVs were quantified 24 h after heat shock, the number of released EVs from heat-stressed WT cultures was significantly lower than that from cultures maintained continuously at 37 °C (Figure 4A). As expected, *Pf*Vps60KO parasites produced fewer EVs under normal conditions, as observed in [30], and their EV abundance remained low following heat shock (Figure 4A).

Previous studies in other cell types have reported increased EV release after heat stress [21,48], contrasting with our observations. These differences suggest that thermal stress responses may differ among organisms, reflecting distinct survival strategies. Given the combination of reduced EV release in WT parasites and the markedly lower viability of the EV-deficient line under heat stress, we considered an alternative explanation: in *P. falciparum*, rather than a sustained increase in EV secretion, febrile stress might trigger rapid EV turnover through enhanced release followed by internalization. Such mechanisms could confer a selective advantage by facilitating the uptake of EV-associated protective or stress-response factors by other neighbouring parasite cells.

To test this hypothesis, EVs were isolated at earlier time points after heat shock exposure. When EV harvesting was performed 2 h after the heat shock, a significant increase in EV abundance was detected in both 3D7 and *Pf*Vps60KO cultures compared with untreated controls (Figure 4B). These results indicate that heat shock transiently promotes EV release in *P. falciparum*, supporting the notion that EV secretion is part of an immediate adaptive response to febrile stress.

### P. falciparum-derived EVs contain aggregation-prone proteins

**Figure 5.**
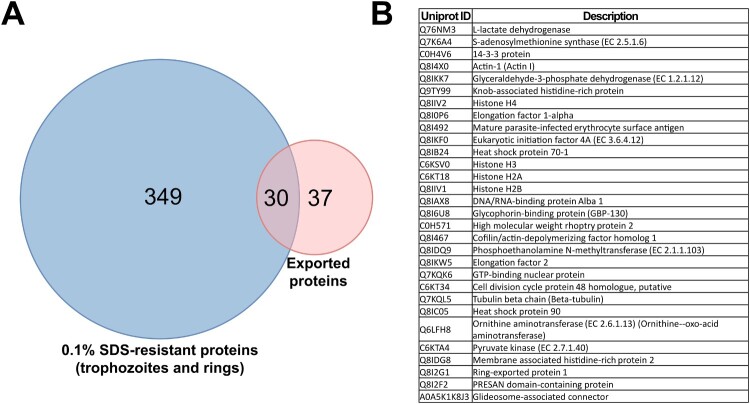
LC-MS/MS data analysis of aggregation-prone proteins found in pRBC lysates and in EVs of the 3D7 line. (A) Venn diagram showing the number of proteins identified in 0.1% SDS-resistant pellets purified from trophozoite and ring stages as well as in *P. falciparum*-derived EVs, and the proteins shared between the two samples. (B) List of aggregation-prone proteins identified in *P. falciparum*-derived EVs, according to TANGO score. Data represents hits detected in at least 3 of 4 biological replicates.

Building on the observed stress sensitivity upon *Pf*Vps60 disruption, we next investigated whether EVs released by *P. falciparum* 3D7 contained aggregation-prone or chaperone-related proteins that could contribute to the parasite’s stress response. Previously, we demonstrated the presence of aggregation-prone proteins in live *P. falciparum* cultures, which accumulated within the parasite cytoplasm throughout its life cycle [49]. However, the export of these protein aggregates via EVs and their potential activity in recipient cells has remained unexplored. To address this, proteins isolated from 3D7-derived EVs were extracted and compared with a previously defined set of aggregative proteins identified in whole-parasite lysates [41]. Across three independent biological replicates, a total of 67 EV-associated proteins were identified, of which 30 (44.8%) overlapped with the aggregation-prone set (Figure 5A, Table S2).

Among the EV-associated proteins were the molecular chaperones *Pf*Hsp70-1 (Q8IB24) and *Pf*Hsp90 (Q8IC05), which form a functional complex involved in protein folding and protection against aggregation [50,51]. In addition, EVs contained aggregation-prone proteins associated with host cell invasion (C0H4V6, Q8I4X0, Q8I6U8), cytoskeletal remodelling (Q7KQL5, Q8IDG8), and gene regulation (C6KSV0, C6KT18, Q8IIV1, Q7KQK6), along with other proteins that may participate in multiprotein complex assemblies (Figure 5B). These findings suggested that *P. falciparum*-derived EVs contain aggregation-prone and proteostasis-related proteins, supporting a role for EVs during proteotoxic stress.

### PfVps60-deficient parasites accumulate aggregative proteins in the vicinity of the parasitophorous vacuole

**Figure 6.**
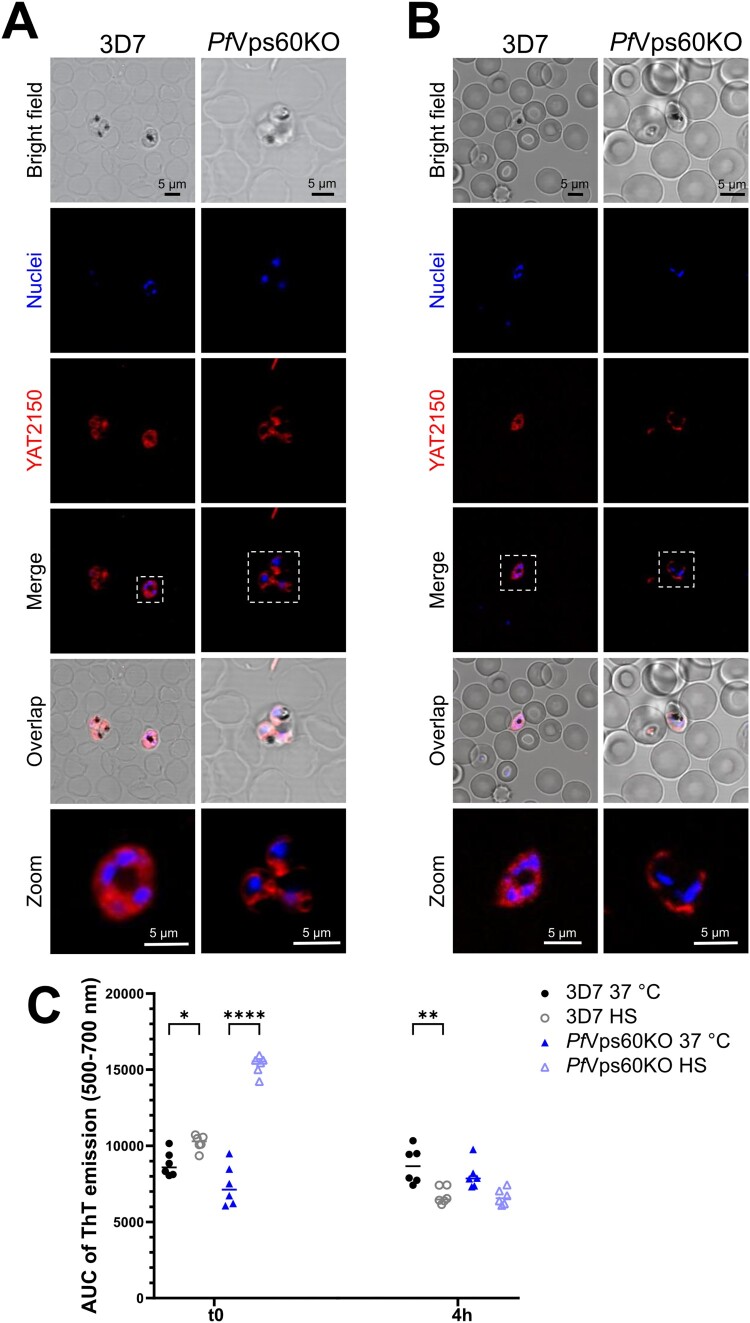
Distribution of aggregative proteins in *P. falciparum* 3D7 and *Pf*Vps60KO strains. Confocal fluorescence microscopy analysis of (A) fixed or (B) live *P. falciparum* blood stages of the 3D7 WT and *Pf*Vps60KO strains stained for 30 min with 0.5 µM YAT2150 (red); nuclei were counterstained with Hoechst 33342 (blue). The bottom panels show zooms of the indicated regions in the merge panels, to allow better visualization. (C) Quantification of aggregation by ThT assay in synchronized trophozoite-stage parasite lysates. Parasites were maintained at 37 °C or exposed to heat shock (HS). Samples were collected immediately after heat shock (t0) or after an additional 4 h recovery period at 37 °C (4 h). Aggregation rates were estimated by calculating the area under the curve (AUC) of the fluorescence emission spectra (500–700 nm). Each symbol represents a biological replicate, and horizontal bars indicate the mean. Statistical analysis was performed using two-way ANOVA with Šídák’s multiple comparisons test. *p ≤ 0.05, **p ≤ 0.01, ****p ≤ 0.0001.

Given that *Pf*Vps60 disruption selectively impaired the EV-associated export of chaperones and other proteins, we next examined whether loss of this protein affects global proteostasis within the parasite. As discussed earlier, *Pf*Hsp110 contributes to *P. falciparum* proteostasis through its ability to bind unfolded proteins via its substrate-binding domain [19]. Since this chaperone was absent from EVs derived from the *Pf*Vps60KO line, we next assessed whether disruption of ESCRT-dependent EV trafficking impacted intracellular protein aggregation. To this end, we performed confocal fluorescence microscopy using YAT2150, a fluorescent probe previously characterized by our group that detects intracellular protein aggregates in *Plasmodium* cells [41,49]. Fixed (Figure 6A) and live (Figure 6B) *P. falciparum* cultures of the WT and the *Pf*Vps60KO strains were analyzed and compared. In WT parasites, protein aggregates displayed a homogenous cytoplasmic distribution. In contrast, *Pf*Vps60-deficient parasites exhibited a distinct localization of the aggregated protein pool toward the periphery of the parasite, accumulating near the parasitophorous vacuole (see zoom panels in Figure 6), a differential distribution particularly evident in live preparations. Importantly, because *Pfvps60* knockout does not alter the parasite’s intraerythrocytic development [30], the observed spatial redistribution of aggregative proteins likely reflected an altered proteostasis response rather than a general defect in the asexual cycle under basal conditions.

To further determine whether this altered spatial distribution of aggregation-prone proteins translated into changes in global proteostasis dynamics, we performed a quantitative analysis of protein aggregation using ThT fluorescence assays, which enables the detection of β-sheet-enriched proteins in the parasite lysate. As observed in Figure 6C, analysis of ThT fluorescence revealed significant differences in protein aggregation between conditions at early time points. Immediately following heat shock (t0), *Pf*Vps60-deficient parasites displayed a marked increase in ThT signal compared to WT and control conditions, indicating an early perturbation of proteostasis. In contrast, 4 h following initial thermal treatment (4 h), WT parasites exhibited a significant reduction in ThT signal under heat shock relative to 37 °C controls, suggesting an active remodelling or clearance of aggregation-prone species. In contrast, *Pf*Vps60KO parasites maintained aggregation levels similar to the control. The marked decrease of ThT signal observed in heat-shocked *Pf*Vps60KO parasites between t0 and 4 h may reflect a combination of stress-induced remodelling of ThT-reactive species and disposal of misfolded proteins during early recovery. Because ThT fluorescence reports β-sheet-enriched species rather than total protein aggregation, this decrease should not be interpreted solely as aggregate clearance.

### Aggregation-prone enriched EVs associate with the surface of naïve RBCs following heat shock

**Figure 7.**
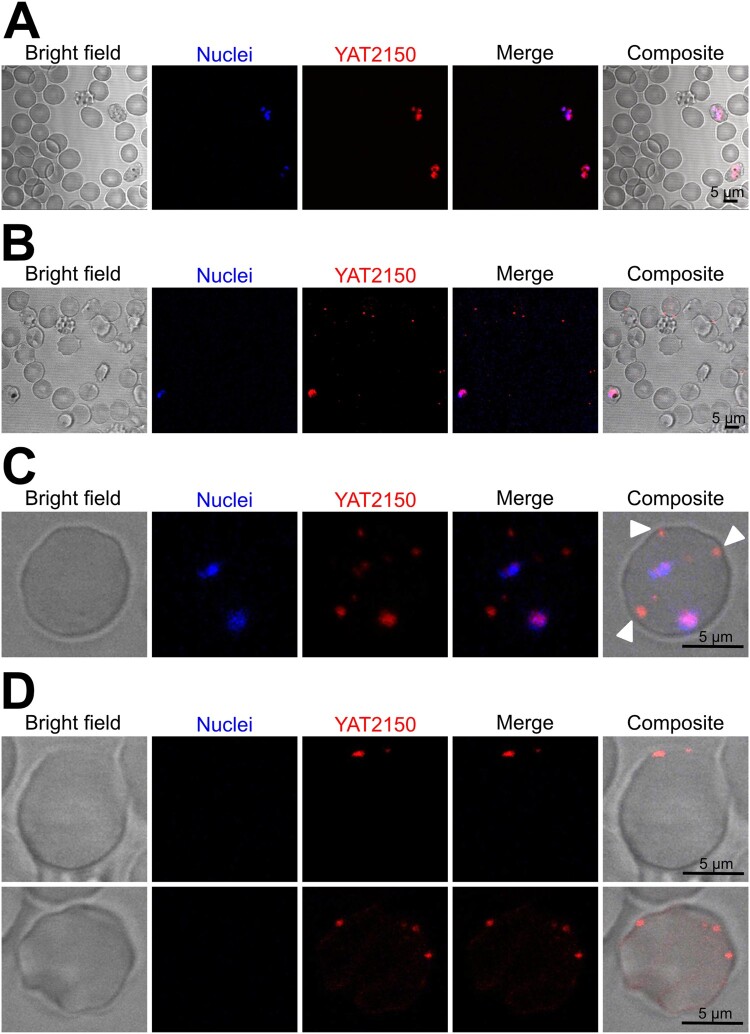
Heat shock-induced EVs associate with neighbouring naïve RBCs. Representative confocal fluorescence microscopy images of (A) 3D7 parasites maintained at 37 °C or (B) subjected to heat shock at 41 °C for 30 min. (C) Detail of an infected RBC showing small vesicles adjacent to the plasma membrane. (D) Representative images showing YAT2150-labelled EVs associated with the surface of naïve RBCs. In all cases, cells were stained with 0.5 µM YAT2150 (red) and nuclei counterstained with Hoechst 33342 (blue).

To investigate whether stress-induced vesicle release influenced neighbouring cells, *P. falciparum* 3D7 parasites were exposed to heat shock and subsequently labelled with YAT2150. Nuclear staining allowed discrimination between infected and uninfected RBCs. In untreated control cultures, no YAT2150-positive vesicles were observed on the surface of neighbouring RBCs (Figure 7A). In contrast, following heat shock treatment, EVs enriched in aggregative proteins (as indicated by YAT2150 fluorescence) accumulated on the surface of adjacent uninfected RBCs (Figure 7B). In some cases, vesicles were detected at the periphery of pRBCs, apparently ready for release into the extracellular space (Figure 7C, arrowheads). High magnification images revealed that vesicles remained attached to the membrane of neighbouring naïve RBCs (Figure 7D), and in certain cells, YAT2150 fluorescence was diffusely distributed throughout the RBC cytoplasm (Figure 7D, lower row), an observation consistent with a possible cargo transfer into the target cell, although direct uptake was not demonstrated. These results indicated that EVs released from heat-stressed parasites can associate with neighbouring erythrocytes and are in agreement with a potential role of EVs in intercellular communication under stress conditions.

### Wild type-derived EVs confer protection to PfVps60KO parasites under heat shock stress

**Figure 8.**
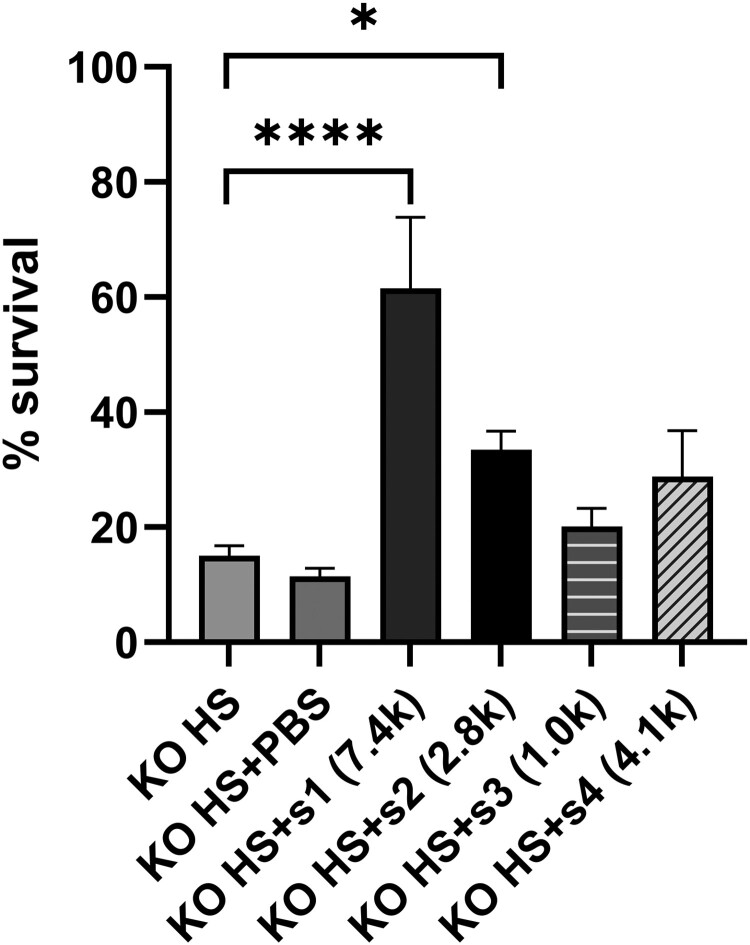
Extracellular vesicles from the 3D7 line rescue *Pf*Vps60KO parasites from heat shock-induced death in a dose-dependent manner. Parasite survival following heat shock (41 °C, 2 h) was quantified by flow cytometry using Syto11. During heat stress, *Pf*Vps60KO parasites were supplemented with three EV preparations derived from heat shocked 3D7 parasites: s1, s2 and s3, as well as one derived from 3D7 parasites maintained at 37 °C (s4). Average EV concentrations are indicated in the x-axis labels (kcps/mL). All EV preparations were added at a fixed volume but contained different EV concentrations (s1 > s4 > s2 > s3). A control containing only PBS was included. Bars represent mean ± SD of three biological replicates. HS: heat shock. Statistical significance was calculated using one-way ANOVA. *p ≤ 0.05, ****p ≤ 0.0001.

Based on the marked heat shock sensitivity of *Pf*Vps60KO parasites detailed in Figure 3, and on the association of EVs enriched in aggregation-prone proteins with neighbouring erythrocytes following thermal stress, we next examined whether extracellular vesicles could functionally restore parasite survival under febrile conditions. For this, *Pf*Vps60KO parasites were exposed to heat shock in the presence of EVs derived from heat shocked 3D7 cultures, which were added immediately prior to and maintained throughout the heat shock period. Three independent EV preparations (s1, s2, and s3), containing decreasing EV concentrations (s1 > s2 > s3) but added at a fixed volume were tested. Supplementation with the most concentrated preparations (s1 and s2) significantly increased survival relative to the heat shocked *Pf*Vps60KO untreated control, whereas the least concentrated sample (s3) did not provide detectable protection (Figure 8). Moreover, an additional EV preparation (s4) derived from 3D7 parasites maintained at 37 °C, despite having high EV abundance, failed to confer a significantly protective effect under heat shock conditions. These results suggested that vesicles released by WT parasites under heat shock conditions contain factors that can partially restore parasite viability in a dose-dependent manner. 1× PBS used as a control in equal tested volumes did not exhibit any significant effect.

## Discussion

In *P. falciparum*, proteostasis represents a major challenge even in the absence of external stress. The parasite’s atypical genome composition (approximately 81% of adenine (A) and thymine (T) residues [52]) results in an extreme A/T bias leading to an overrepresentation of asparagine and glutamine repeats, which constitute nearly 30% of the proteome [53] and are intrinsically prone to misfolding and aggregation [54,55]. This aggregation-prone nature has been confirmed through the isolation of 0.1% SDS-resistant aggregates from parasite cultures, as well as by staining with the commercial kit PROTEOSTAT®, which specifically detects protein aggregates in live cells [49]. In addition, the active ingredient of PROTEOSTAT®, YAT2150, synthesized in-house, stained aggregates formed *in vitro* by aggregation-prone peptides selected from the *P. falciparum* proteome [49,56], and in-cell throughout all the blood stages of the parasite [41]. These intrinsic features indicated that *P. falciparum* is particularly dependent on efficient protein quality control mechanisms.

To cope with this aggregation-prone proteome and the recurrent temperature fluctuations experienced during infection, *Plasmodium* has evolved a highly effective heat shock defense system. More than 90 Hsp have been identified in the parasite, playing key roles during development, survival, and stress adaptation [57]. Unlike other eukaryotes, some *Plasmodium* Hsp are constitutively expressed at high levels [19,58,59], but also upregulated upon heat shock [58–60], hence combining house-keeping and stress response roles. Several Hsp are essential for parasite survival, especially members of the Hsp90, Hsp70, and Hsp40 families [61–63]. These chaperones can operate both in functional networks and independently with their corresponding co-chaperones [64–67]. Moreover, Hsp are widely distributed in all the parasite organelles and in the host, where they exhibit different functions. For instance, *Pf*Hsp70-1 (Q8IB24) is the major cytosolic chaperone expressed during the intraerythrocytic life cycle, it is upregulated upon heat shock and participates in the maintenance of proteostasis [45]. As occurs in higher eukaryotes, some *Plasmodium* Hsp are exported to the extracellular space through the endomembrane system, such as *Pf*Hsp70-x (K7NTP5) [68] and *Pf*Hsp40 (O96123) [69], which could participate in systemic stress responses.

In this study, we show that disruption of *Pf*Vps60 impairs EV-associated export of the chaperones *Pf*Hsp70-x and *Pf*Hsp110. Both are known to assist in protein folding and to protect the parasite from the aggregation driven by its low-complexity proteome, as well as from temperature fluctuations experienced throughout its life cycle [19,66]. The absence of these chaperones from EVs derived from the *Pf*Vps60KO strain suggests that the ESCRT-mediated vesicular trafficking contributes to their export, either through EV biogenesis or cargo selection. This observation is consistent with our previous work showing that *Pf*Vps60 disruption reduces EV production [30]. The functional consequences of these defects are evident under thermal stress. In particular, induction of the major cytosolic chaperone *Pf*Hsp70-1, which peaked coinciding with nuclear division at the end of the trophozoite stage, was significantly weaker in heat-shocked *Pf*Vps60KO compared to heat-shocked WT parasites. Given that *Pf*Hsp70-1 is strongly transcriptionally induced during heat shock, with reported increases up to ∼16-fold mediated by the *Pf*AP2-HS transcription factor [36], the reduced induction observed in *Pf*Vps60KO parasites may reflect both potential alterations in stress-responsive transcriptional programmes and impaired proteostasis. Consistent with this, YAT2150 staining showed an evident accumulation of aggregation-prone proteins near the parasitophorous vacuole in *Pf*Vps60-deficient parasites, which exhibited markedly impaired recovery from thermal stress. The redistribution of aggregates toward the parasitophorous vacuole suggests that defective vesicular export may contribute to local accumulation of misfolded proteins at sites normally involved in EV budding [70]. Complementary ThT measurements further revealed an early increase followed by a decrease in signal in *Pf*Vps60KO parasites after heat shock. Because ThT selectively detects β-sheet-rich species, this decline likely reflects remodelling of ThT-reactive aggregates and/or loss of severely damaged proteins, rather than efficient aggregate clearance.

Previously, it has been proposed that EVs released in response to stress can trigger intercellular signalling in neighbouring cells while simultaneously enhancing their resilience to subsequent challenges (bystander effect) [21]. Although this specific condition was not directly tested here, our observations align with this model. We found that EV production is dynamically regulated in response to heat shock with a rapid increase shortly after stress exposure followed by a decrease 24 h post-stress, indicating that EV release constitutes an early and transient adaptive response rather than a sustained secretory process. It is important to note that EV quantification in this study is based on DLS-derived particle counts, which provide a relative rather than absolute measurement and do not directly assess vesicle purity. Similar stress-dependent modulation of EV cargo and abundance and cargo composition has been reported under nutrient starvation, where *P. falciparum* exhibited a rapid increase in EV release together with altered protein and small RNA composition, particularly in ring-stage cultures [71], supporting the notion that EV-mediated responses may represent a conserved mechanism in malaria parasites.

Consistent with this model, EVs containing aggregation-prone cargoes were observed adhering to neighbouring uninfected erythrocytes following heat shock, indicating that stress-induced EVs can interact with surrounding cells. However, direct evidence of cargo transfer and functional uptake remains to be established, and the precise mechanisms governing EV trafficking, uptake, and cargo delivery between infected and uninfected erythrocytes require further investigation. Likewise, although our data reveal enrichment of aggregation-prone proteins and molecular chaperones in EVs, the specific contribution of individual cargo components is yet unresolved. Our findings suggest that the protective effect of EVs likely arises from the combined action of multiple stress-related factors, and dissecting the relative contribution of individual cargo components will require targeted functional approaches in future work.

We provide functional evidence that EVs released by WT parasites under heat shock conditions can partially protect *Pf*Vps60-deficient parasites from severe thermal sensitivity in a dose-dependent manner when administered during the heat shock period. This experimental design aligns with a role for EVs in acute stress protection rather than preconditioning or post-stress recovery, since EVs obtained under non-stressed conditions fail to confer protection. These findings indicate that stress-induced EV cargoes are associated with improved parasite survival under heat shock conditions and may contribute to the restoration of proteostasis-related functions.

The present study has some limitations that should be considered when interpreting the results. First, all experiments were performed using *in vitro* culture systems, which may not fully mimic the complexity of *in vivo* infection dynamics during clinical malaria. Second, our analyses were performed in a single parasite genetic background (3D7), and strain-specific differences in stress responses or EV biology cannot be excluded. Finally, while our findings support a role for EV-associated cargo in stress adaptation, *in vivo* validation will be required to establish the physiological relevance of this pathway during infection.

Taken together, our results identify ESCRT-dependent EV trafficking as a key component of the heat shock response in *P. falciparum*, linking proteostasis, vesicular communication, and parasite survival under febrile conditions. EVs may contribute to the redistribution of aggregation-prone proteins and molecular chaperones during stress, as well as to coordinated adaptation within the parasite population. These findings suggest that EV biogenesis may represent a druggable vulnerability, raising the possibility that interfering with this pathway could sensitize parasites to febrile stress, and representing a novel strategy to disrupt *Plasmodium* survival during clinically relevant febrile episodes.

## Supplementary Material

Supplemental Material

## Data Availability

All relevant data are within the manuscript and its Supporting Information files.
