# Supplementary material for: Extracellular vesicles participate in proteostasis and heat shock adaptation in Plasmodium falciparum
Source: Emerg Microbes Infect. 2026 Jun 8;15(1):2686468. doi: 10.1080/22221751.2026.2686468 (PMC13312847; doi:10.1080/22221751.2026.2686468)
Supplement: Supplemental Material [file TEMI_A_2686468_SM1867.docx]

**Extracellular vesicles participate in proteostasis and heat shock adaptation in *Plasmodium falciparum***

Yunuen Avalos-Padilla^1,2,*^, Lucía Román-Álamo^1,2^, Inés Bouzón-Arnáiz^1,2^, Elsa M. Arce^3^, Diego Muñoz-Torrero^3,4^ and Xavier Fernàndez-Busquets^1,2,5,*^.

^1^Barcelona Institute for Global Health (ISGlobal), Hospital Clínic-Universitat de Barcelona, Barcelona, Spain.

^2^Nanomalaria Group, Institute for Bioengineering of Catalonia (IBEC), The Barcelona Institute of Science and Technology, Barcelona, Spain.

^3^Laboratory of Medicinal Chemistry, Faculty of Pharmacy and Food Sciences, University of Barcelona, Barcelona, Spain.

^4^Institute of Biomedicine (IBUB), University of Barcelona, Barcelona, Spain

^5^Nanoscience and Nanotechnology Institute (IN2UB), University of Barcelona, Barcelona, Spain.

*Corresponding authors

Email: [yavalos@ibecbarcelona.eu](mailto:yavalos@ibecbarcelona.eu) (YA-P), [xfernandez@ibecbarcelona.eu](mailto:xfernandez@ibecbarcelona.eu) (XF-B)

**Supplementary materials**


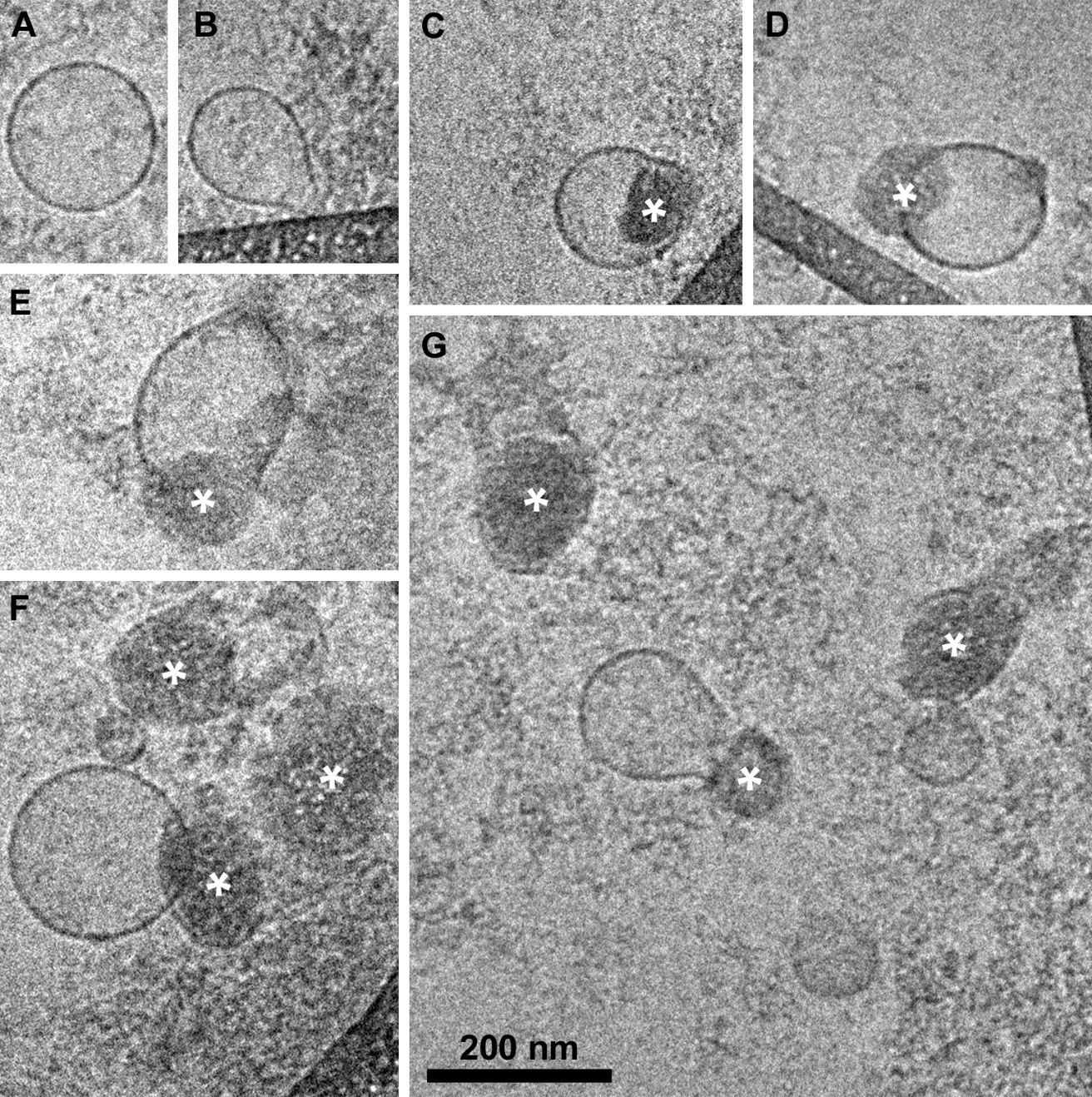


**Figure S1. Representative cryoTEM micrographs of EV preparations obtained from *Pf*Vps60KO-infected erythrocyte cultures.** Structures of morphology consistent with extracellular vesicles were observed, including heterogeneous populations with variable electron density. (A) Example of vesicle without dense inclusions. (B) Example of ruptured vesicle adjacent to electron-dense extravesicular material. (C-E) Examples of ruptured vesicles partially containing electron-dense material. (F,G) Fields containing different types of vesicles. Asterisks indicate electron-dense intravesicular material or material associated with partially disrupted vesicles.


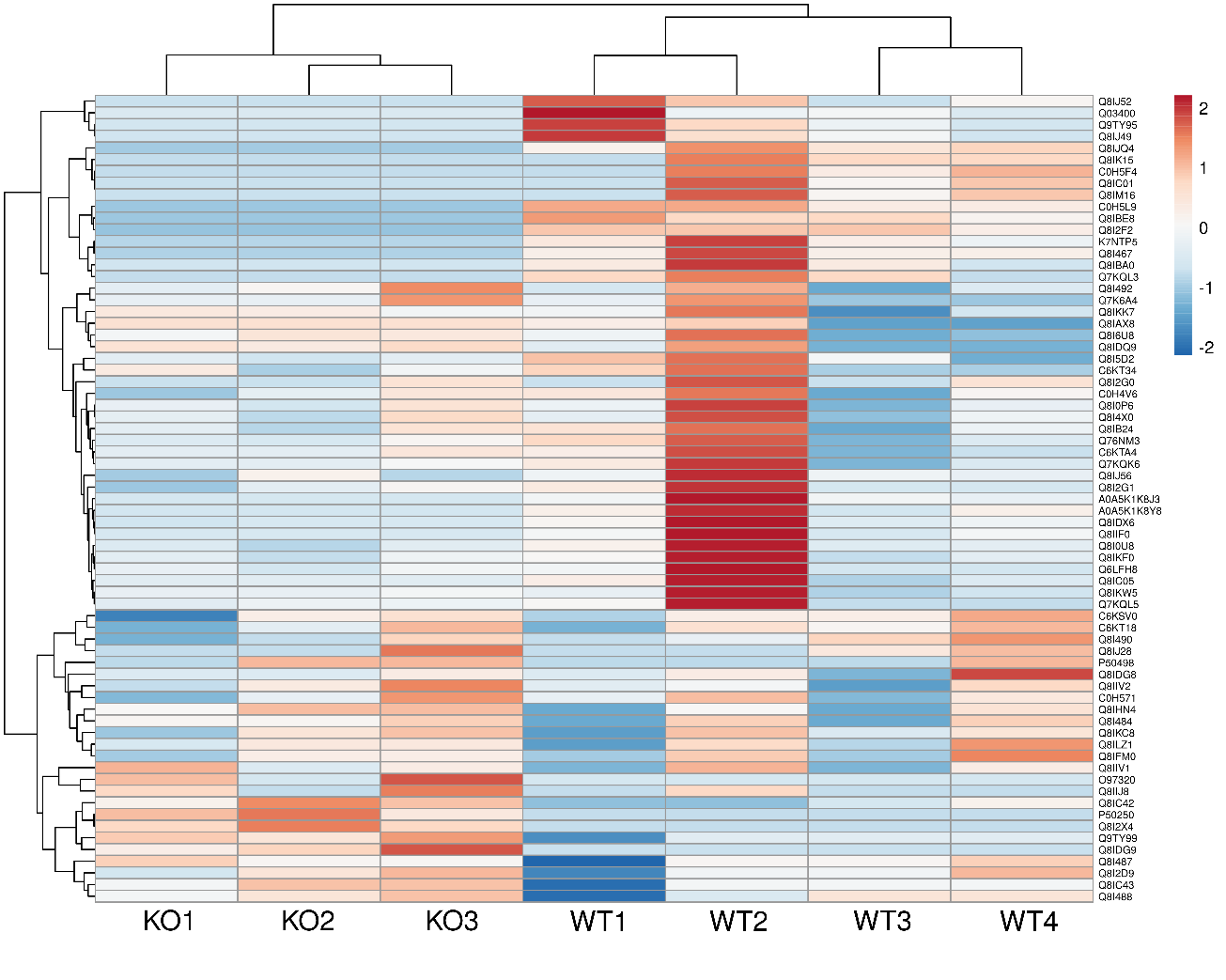


**Figure S2. Heatmap of EV-associated protein abundance in WT and *Pf*Vps60KO parasites.** Relative protein abundance of the 69 *P. falciparum* proteins detected across biological replicates of the different strains is shown. Data were log2-transformed, mean-centered and scaled by rows (unit variance scaling). Hierarchical clustering was performed using Euclidean distance and average linkage. Each row represents a protein (Uniprot accession), and each column corresponds to an individual sample.


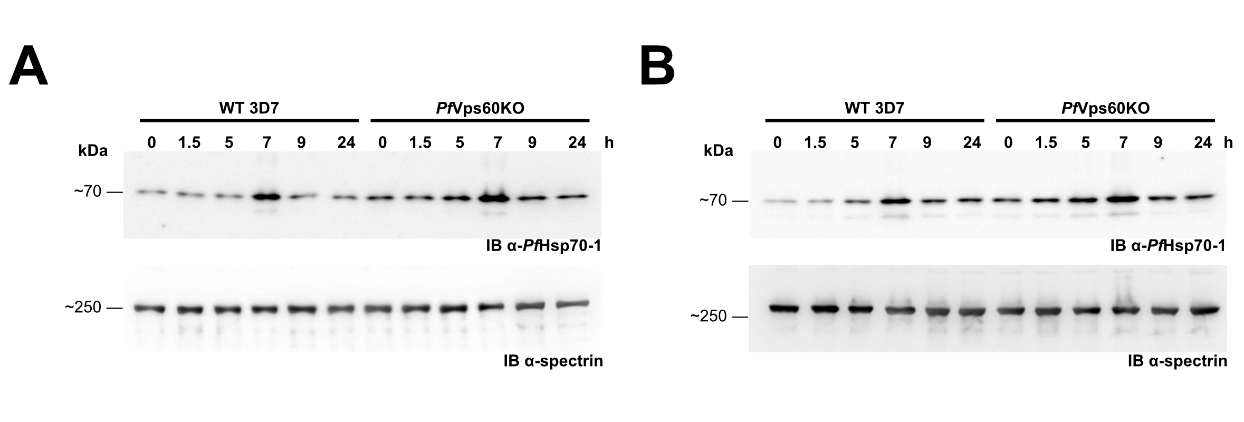


**Figure S3. Representative Western blot** **images** **for** ***Pf*Hsp70-1 quantification.** Representative blots corresponding to the quantifications shown in Figure 2A and 2B, depicting *Pf*Hsp70-1 (~70 kDa) and spectrin (~250 kDa) levels in WT and *Pf*Vps60KO samples under control conditions (37 °C; panel A) and heat shock conditions (41 °C; panel B). Samples were collected at the indicated time points (0, 1.5, 5, 7, 9 and 24 h). Spectrin was used as a loading control. IB: immunoblot.

**Table S1. List of *P. falciparum* proteins identified by LC-MS/MS in extracellular vesicles from 3D7 and *Pf*Vps60KO strains.** Boxes in darker gray highlight proteins only found in the 3D7 sample, whereas those in lighter gray indicate proteins only found in the *Pf*Vps60KO strain. In light orange the protein *Pf*Hsp70-1 whose export is not altered in the mutant strain. Protein identification was accepted at a false discovery rate (FDR) ≤1% using Percolator-based filtering. Proteins were retained for analysis if detected in at least 3 out of 4 biological replicates for WT samples and at least 2 out of 3 biological replicates for *Pf*Vps60KO samples. Σ#PSMs: total number of peptide-spectrum matches across all replicates. #PSM: number of peptide-spectrum matches detected per biological replicate. AAs: total number of amino acids of the entire sequence of the protein. MW: molecular weight; calc. pI: calculated isoelectric point.

|  |  |  |  | **KO1** | **KO2** | **KO3** | **WT1** | **WT2** | **WT3** | **WT4** |  |  |  |
| --- | --- | --- | --- | --- | --- | --- | --- | --- | --- | --- | --- | --- | --- |
| **Accession (Uniprot)** | **Accession (PlasmoDB)** | **Description** | **Σ# PSMs** | **# PSM KO1** | **# PSM KO2** | **# PSM KO3** | **# PSM WT1** | **# PSM WT2** | **# PSM WT3** | **# PSM WT4** | **# AAs** | **MW [kDa]** | **calc. pI** |
| Q8I0U8 | PF3D7_0930300 | Merozoite surface antigens OS=Plasmodium falciparum (isolate 3D7) OX=36329 GN=PF3D7_0930300 PE=4 SV=1 - [Q8I0U8_PLAF7] | 420 | 47 | 36 | 50 | 66 | 122 | 48 | 51 | 1720 | 195.6 | 6.51 |
| Q8IKK7 | PF3D7_1462800 | Glyceraldehyde-3-phosphate dehydrogenase OS=Plasmodium falciparum (isolate 3D7) OX=36329 GN=PF3D7_1462800 PE=3 SV=1 - [Q8IKK7_PLAF7] | 213 | 35 | 34 | 30 | 30 | 47 | 13 | 24 | 337 | 36.6 | 7.69 |
| Q8I0P6 | PF3D7_1357000 | Elongation factor 1-alpha OS=Plasmodium falciparum (isolate 3D7) OX=36329 GN=PF3D7_1357000 PE=3 SV=1 - [Q8I0P6_PLAF7] | 199 | 26 | 22 | 34 | 27 | 48 | 16 | 26 | 443 | 48.9 | 9.06 |
| Q76NM3 | PF3D7_1324900 | L-lactate dehydrogenase OS=Plasmodium falciparum (isolate 3D7) OX=36329 GN=PF3D7_1324900 PE=1 SV=1 - [Q76NM3_PLAF7] | 154 | 18 | 17 | 23 | 29 | 38 | 11 | 18 | 316 | 34.1 | 7.55 |
| Q8I492 | PF3D7_0500800 | Mature parasite-infected erythrocyte surface antigen OS=Plasmodium falciparum (isolate 3D7) OX=36329 GN=PF3D7_0500800 PE=4 SV=1 - [Q8I492_PLAF7] | 142 | 18 | 21 | 31 | 16 | 29 | 10 | 17 | 1434 | 168.2 | 4.78 |
| Q8IJ56 | PF3D7_1035300 | Glutamate-rich protein GLURP OS=Plasmodium falciparum (isolate 3D7) OX=36329 GN=PF3D7_1035300 PE=4 SV=1 - [Q8IJ56_PLAF7] | 131 | 12 | 20 | 13 | 18 | 33 | 18 | 17 | 1233 | 141.0 | 4.32 |
| Q8I6U8 | PF3D7_1016300 | Glycophorin-binding protein OS=Plasmodium falciparum (isolate 3D7) OX=36329 GN=GBP PE=3 SV=1 - [GBP_PLAF7] | 126 | 18 | 23 | 22 | 17 | 34 | 5 | 7 | 824 | 95.8 | 5.14 |
| C6KTA4 | PF3D7_0626800 | Pyruvate kinase OS=Plasmodium falciparum (isolate 3D7) OX=36329 GN=PF3D7_0626800 PE=1 SV=1 - [C6KTA4_PLAF7] | 97 | 10 | 10 | 19 | 17 | 35 |  | 6 | 511 | 55.6 | 7.55 |
| Q8I4X0 | PF3D7_1246200 | Actin-1 OS=Plasmodium falciparum (isolate 3D7) OX=36329 GN=PFL2215w PE=1 SV=1 - [ACT1_PLAF7] | 96 | 12 | 9 | 18 | 12 | 25 | 7 | 13 | 376 | 41.8 | 5.34 |
| Q8IDX6 | PF3D7_1335400 | Reticulocyte-binding protein 2 homolog a OS=Plasmodium falciparum (isolate 3D7) OX=36329 GN=PF13_0198 PE=1 SV=1 - [RBP2A_PLAF7] | 88 | 2 | 4 | 4 | 14 | 47 | 6 | 11 | 3130 | 370.2 | 5.60 |
| Q8IB24 | PF3D7_0818900 | Heat shock protein 70-1 OS=Plasmodium falciparum (isolate 3D7) OX=36329 GN=PF3D7_0818900 PE=3 SV=1 - [Q8IB24_PLAF7] | 76 | 9 | 6 | 14 | 14 | 20 | 3 | 10 | 677 | 73.9 | 5.67 |
| Q8IC43 | PF3D7_0702400 | Small exported membrane protein 1 OS=Plasmodium falciparum (isolate 3D7) OX=36329 GN=PF3D7_0702400 PE=4 SV=1 - [Q8IC43_PLAF7] | 63 | 9 | 11 | 11 | 5 | 9 | 9 | 9 | 123 | 14.2 | 9.60 |
| C0H5F4 | PF3D7_1335300 | Reticulocyte binding protein 2 homolog b OS=Plasmodium falciparum (isolate 3D7) OX=36329 GN=Rh2b PE=1 SV=1 - [RBP2B_PLAF7] | 59 |  |  |  |  | 26 | 12 | 21 | 3179 | 374.0 | 5.27 |
| Q8IC05 | PF3D7_0708400 | Heat shock protein 90 OS=Plasmodium falciparum (isolate 3D7) OX=36329 GN=PF3D7_0708400 PE=1 SV=1 - [Q8IC05_PLAF7] | 59 | 5 | 5 | 5 | 11 | 29 |  | 4 | 745 | 86.1 | 5.01 |
| Q8IIV2 | PF3D7_1105000 | Histone H4 OS=Plasmodium falciparum (isolate 3D7) OX=36329 GN=PF3D7_1105000 PE=3 SV=1 - [Q8IIV2_PLAF7] | 56 | 6 | 9 | 12 | 7 | 8 | 4 | 10 | 103 | 11.4 | 11.22 |
| Q8IKW5 | PF3D7_1451100 | Elongation factor 2 OS=Plasmodium falciparum (isolate 3D7) OX=36329 GN=PF3D7_1451100 PE=3 SV=1 - [Q8IKW5_PLAF7] | 56 | 6 | 6 | 8 | 6 | 28 |  | 2 | 832 | 93.5 | 6.80 |
| Q8IKC8 | PF3D7_1471100 | Exported protein 2 OS=Plasmodium falciparum (isolate 3D7) OX=36329 GN=PF3D7_1471100 PE=1 SV=1 - [Q8IKC8_PLAF7] | 56 | 6 | 9 | 10 | 5 | 10 | 7 | 9 | 287 | 33.4 | 5.27 |
| C0H4V6 | PF3D7_0818200 | 14-3-3 protein OS=Plasmodium falciparum (isolate 3D7) OX=36329 GN=PF3D7_0818200 PE=3 SV=1 - [C0H4V6_PLAF7] | 54 | 5 | 7 | 9 | 9 | 12 | 4 | 8 | 262 | 30.2 | 4.92 |
| Q7KQK6 | PF3D7_1117700 | GTP-binding nuclear protein OS=Plasmodium falciparum (isolate 3D7) OX=36329 GN=PF3D7_1117700 PE=3 SV=1 - [Q7KQK6_PLAF7] | 51 | 5 | 5 | 7 | 9 | 18 |  | 4 | 214 | 24.9 | 7.94 |
| Q8IDQ9 | PF3D7_1343000 | Phosphoethanolamine N-methyltransferase OS=Plasmodium falciparum (isolate 3D7) OX=36329 GN=PF3D7_1343000 PE=4 SV=1 - [Q8IDQ9_PLAF7] | 49 | 10 | 9 | 11 | 5 | 14 |  |  | 266 | 31.0 | 5.60 |
| Q8ILZ1 | PF3D7_1410400 | Rhoptry-associated protein 1 OS=Plasmodium falciparum (isolate 3D7) OX=36329 GN=PF3D7_1410400 PE=4 SV=1 - [Q8ILZ1_PLAF7] | 47 | 5 | 8 | 8 | 2 | 9 | 4 | 11 | 782 | 90.0 | 7.11 |
| Q8I2G1 | PF3D7_0935900 | Ring-exported protein 1 OS=Plasmodium falciparum (isolate 3D7) OX=36329 GN=PF3D7_0935900 PE=4 SV=1 - [Q8I2G1_PLAF7] | 45 | 2 | 5 | 7 | 8 | 15 | 4 | 4 | 713 | 83.0 | 5.59 |
| C6KSV0 | PF3D7_0610400 | Histone H3 OS=Plasmodium falciparum (isolate 3D7) OX=36329 GN=PF3D7_0610400 PE=3 SV=1 - [C6KSV0_PLAF7] | 44 |  | 7 | 8 | 3 | 7 | 7 | 10 | 136 | 15.4 | 11.14 |
| Q8I488 | PF3D7_0501200 | Parasite-infected erythrocyte surface protein OS=Plasmodium falciparum (isolate 3D7) OX=36329 GN=PF3D7_0501200 PE=4 SV=1 - [Q8I488_PLAF7] | 42 | 6 | 7 | 8 | 2 | 5 | 7 | 7 | 408 | 48.7 | 7.18 |
| K7NTP5 | PF3D7_0831700 | Heat shock protein 70 OS=Plasmodium falciparum (isolate 3D7) OX=36329 GN=PF3D7_0831700 PE=1 SV=1 - [K7NTP5_PLAF7] | 42 |  |  |  | 9 | 20 | 8 | 5 | 679 | 75.0 | 5.77 |
| C6KT18 | PF3D7_0617800 | Histone H2A OS=Plasmodium falciparum (isolate 3D7) OX=36329 GN=PF3D7_0617800 PE=3 SV=1 - [C6KT18_PLAF7] | 40 | 3 | 5 | 8 | 3 | 7 | 6 | 8 | 132 | 14.1 | 10.29 |
| Q8I490 | PF3D7_0501000 | Uncharacterized protein OS=Plasmodium falciparum (isolate 3D7) OX=36329 GN=PF3D7_0501000 PE=4 SV=1 - [Q8I490_PLAF7] | 38 | 3 | 4 | 7 | 4 | 5 | 7 | 8 | 260 | 30.7 | 9.45 |
| Q8IAX8 | PF3D7_0814200 | DNA/RNA-binding protein Alba 1 OS=Plasmodium falciparum (isolate 3D7) OX=36329 GN=PF3D7_0814200 PE=4 SV=1 - [Q8IAX8_PLAF7] | 35 | 7 | 7 | 7 | 6 | 8 |  |  | 248 | 27.2 | 10.58 |
| Q8IC42 | PF3D7_0702500 | Uncharacterized protein OS=Plasmodium falciparum (isolate 3D7) OX=36329 GN=PF3D7_0702500 PE=4 SV=1 - [Q8IC42_PLAF7] | 30 | 5 | 10 | 8 |  |  | 2 | 5 | 253 | 27.7 | 8.56 |
| Q03400 | PF3D7_1035200 | S-antigen protein OS=Plasmodium falciparum (isolate 3D7) OX=36329 GN=PF10_0343 PE=2 SV=2 - [SANT_PLAF7] | 29 |  |  |  | 22 | 3 | 4 |  | 585 | 62.9 | 4.40 |
| Q8I5D2 | PF3D7_1228600 | 101 kDa malaria antigen OS=Plasmodium falciparum (isolate 3D7) OX=36329 GN=ABRA PE=1 SV=1 - [ABRA_PLAF7] | 28 | 3 | 2 | 3 | 7 | 9 | 4 |  | 743 | 86.6 | 4.86 |
| Q7K6A4 | PF3D7_0922200 | S-adenosylmethionine synthase OS=Plasmodium falciparum (isolate 3D7) OX=36329 GN=PF3D7_0922200 PE=3 SV=1 - [Q7K6A4_PLAF7] | 27 | 3 | 3 | 9 | 3 | 9 |  |  | 402 | 44.8 | 6.74 |
| Q9TY99 | PF3D7_0202000 | Knob-associated histidine-rich protein OS=Plasmodium falciparum (isolate 3D7) OX=36329 GN=PF3D7_0202000 PE=4 SV=1 - [Q9TY99_PLAF7] | 27 | 6 | 5 | 7 |  | 3 | 3 | 3 | 654 | 71.3 | 9.09 |
| Q7KQL5 | PF3D7_1008700 | Tubulin beta chain OS=Plasmodium falciparum (isolate 3D7) OX=36329 GN=PF10_0084 PE=3 SV=1 - [TBB_PLAF7] | 26 | 2 | 3 | 3 | 4 | 14 |  |  | 445 | 49.7 | 4.83 |
| C0H571 | PF3D7_0929400 | High molecular weight rhoptry protein 2 OS=Plasmodium falciparum (isolate 3D7) OX=36329 GN=PF3D7_0929400 PE=4 SV=1 - [C0H571_PLAF7] | 26 |  | 3 | 8 | 3 | 7 |  | 5 | 1378 | 162.6 | 8.27 |
| Q8IKF0 | PF3D7_1468700 | Eukaryotic initiation factor 4A OS=Plasmodium falciparum (isolate 3D7) OX=36329 GN=PF3D7_1468700 PE=3 SV=1 - [Q8IKF0_PLAF7] | 25 | 2 |  | 3 | 4 | 14 |  | 2 | 398 | 45.3 | 5.69 |
| Q8IDG8 | PF3D7_1353200 | Membrane associated histidine-rich protein 2 OS=Plasmodium falciparum (isolate 3D7) OX=36329 GN=PF3D7_1353200 PE=4 SV=1 - [Q8IDG8_PLAF7] | 25 | 3 | 3 | 4 | 3 | 4 | 2 | 6 | 137 | 15.8 | 7.37 |
| Q8IIV1 | PF3D7_1105100 | Histone H2B OS=Plasmodium falciparum (isolate 3D7) OX=36329 GN=PF3D7_1105100 PE=3 SV=1 - [Q8IIV1_PLAF7] | 25 | 5 | 3 | 4 | 2 | 5 | 2 | 4 | 117 | 13.1 | 10.26 |
| Q8I2G0 | PF3D7_0936000 | Ring-exported protein 2 OS=Plasmodium falciparum (isolate 3D7) OX=36329 GN=PF3D7_0936000 PE=4 SV=2 - [Q8I2G0_PLAF7] | 25 | 3 | 3 | 4 | 3 | 5 | 3 | 4 | 94 | 10.8 | 7.87 |
| A0A5K1K8J3 | PF3D7_1361800 | Glideosome-associated connector OS=Plasmodium falciparum (isolate 3D7) OX=36329 GN=PF3D7_1361800 PE=4 SV=1 - [A0A5K1K8J3_PLAF7] | 24 |  |  |  | 3 | 16 | 3 | 2 | 2605 | 290.8 | 5.25 |
| Q6LFH8 | PF3D7_0608800 | Ornithine aminotransferase OS=Plasmodium falciparum (isolate 3D7) OX=36329 GN=OAT PE=1 SV=1 - [OAT_PLAF7] | 23 | 2 |  | 3 | 3 | 15 |  |  | 414 | 46.0 | 6.89 |
| Q8IJQ4 | PF3D7_1014100 | Merozoite surface protein MSA180 OS=Plasmodium falciparum (isolate 3D7) OX=36329 GN=PF3D7_1014100 PE=4 SV=1 - [Q8IJQ4_PLAF7] | 23 |  |  |  | 4 | 8 | 5 | 6 | 1455 | 173.2 | 6.11 |
| Q8I2D9 | PF3D7_0113900 | CX3CL1-binding protein 1 OS=Plasmodium falciparum (isolate 3D7) OX=36329 GN=PF3D7_0113900 PE=4 SV=1 - [Q8I2D9_PLAF7] | 22 | 2 | 4 | 5 |  | 3 | 3 | 5 | 243 | 28.2 | 8.90 |
| O97320 | PF3D7_0320900 | Histone H2A OS=Plasmodium falciparum (isolate 3D7) OX=36329 GN=PF3D7_0320900 PE=3 SV=1 - [O97320_PLAF7] | 20 | 4 |  | 6 |  |  |  |  | 158 | 16.4 | 10.62 |
| Q8IHN4 | PF3D7_1149000 | Antigen 332, DBL-like protein OS=Plasmodium falciparum (isolate 3D7) OX=36329 GN=PF3D7_1149000 PE=4 SV=2 - [Q8IHN4_PLAF7] | 20 | 3 | 5 | 5 |  | 3 |  | 4 | 6093 | 688.9 | 3.89 |
| Q8I487 | PF3D7_0501300 | Protein phosphatase OS=Plasmodium falciparum (isolate 3D7) OX=36329 GN=PF3D7_0501300 PE=3 SV=1 - [Q8I487_PLAF7] | 20 | 4 | 3 | 3 |  | 3 | 3 | 4 | 337 | 36.3 | 4.45 |
| Q9TY95 | PF3D7_0207600 | Serine-repeat antigen protein 5 OS=Plasmodium falciparum (isolate 3D7) OX=36329 GN=SERA5 PE=1 SV=1 - [SERA5_PLAF7] | 19 |  |  |  | 11 | 6 | 2 |  | 997 | 111.7 | 5.41 |
| P50250 | PF3D7_0520900 | Adenosylhomocysteinase OS=Plasmodium falciparum (isolate 3D7) OX=36329 GN=PFE1050w PE=1 SV=2 - [SAHH_PLAF7] | 18 | 6 | 8 | 4 |  |  |  |  | 479 | 53.8 | 5.92 |
| C0H5L9 | PF3D7_1370300 | Membrane associated histidine-rich protein 1 OS=Plasmodium falciparum (isolate 3D7) OX=36329 GN=PF3D7_1370300 PE=4 SV=1 - [C0H5L9_PLAF7] | 16 |  |  |  | 5 | 5 | 3 | 3 | 249 | 28.9 | 6.42 |
| C6KT34 | PF3D7_0619400 | Cell division cycle protein 48 homologue, putative OS=Plasmodium falciparum (isolate 3D7) OX=36329 GN=PF3D7_0619400 PE=4 SV=1 - [C6KT34_PLAF7] | 15 | 3 |  | 2 | 4 | 6 |  |  | 828 | 92.3 | 5.08 |
| A0A5K1K8Y8 | PF3D7_1347500 | DNA/RNA-binding protein Alba 4 OS=Plasmodium falciparum (isolate 3D7) OX=36329 GN=PF3D7_1347500 PE=4 SV=1 - [A0A5K1K8Y8_PLAF7] | 15 |  |  |  | 3 | 9 |  | 3 | 372 | 42.1 | 7.50 |
| Q8IIF0 | PF3D7_1121600 | Exported protein 1 OS=Plasmodium falciparum (isolate 3D7) OX=36329 GN=PF3D7_1121600 PE=4 SV=1 - [Q8IIF0_PLAF7] | 14 |  |  |  | 2 | 10 |  | 2 | 162 | 17.3 | 5.92 |
| Q8IJ49 | PF3D7_1035900 | PUM-HD domain-containing protein OS=Plasmodium falciparum (isolate 3D7) OX=36329 GN=PF3D7_1035900 PE=4 SV=1 - [Q8IJ49_PLAF7] | 14 |  |  |  | 8 | 4 | 2 |  | 566 | 65.1 | 3.92 |
| Q8I484 | PF3D7_0501600 | Rhoptry-associated protein 2 OS=Plasmodium falciparum (isolate 3D7) OX=36329 GN=PF3D7_0501600 PE=4 SV=1 - [Q8I484_PLAF7] | 13 | 2 | 2 | 3 |  | 3 |  | 3 | 398 | 46.7 | 8.79 |
| Q8IBE8 | PF3D7_0731500 | Erythrocyte binding antigen-175 OS=Plasmodium falciparum (isolate 3D7) OX=36329 GN=PF3D7_0731500 PE=1 SV=2 - [Q8IBE8_PLAF7] | 12 |  |  |  | 4 | 3 | 3 | 2 | 1502 | 174.5 | 5.74 |
| Q8I467 | PF3D7_0503400 | Cofilin/actin-depolymerizing factor homolog 1 OS=Plasmodium falciparum (isolate 3D7) OX=36329 GN=PFE0165w PE=1 SV=1 - [CADF1_PLAF7] | 11 |  |  |  | 2 | 5 | 2 | 2 | 122 | 13.7 | 7.78 |
| Q8IFM0 | PF3D7_0424600 | PRESAN domain-containing protein OS=Plasmodium falciparum (isolate 3D7) OX=36329 GN=PF3D7_0424600 PE=4 SV=1 - [Q8IFM0_PLAF7] | 11 |  | 2 | 2 |  | 3 |  | 4 | 309 | 35.9 | 8.57 |
| Q8I2F2 | PF3D7_0936800 | PRESAN domain-containing protein OS=Plasmodium falciparum (isolate 3D7) OX=36329 GN=PF3D7_0936800 PE=1 SV=1 - [Q8I2F2_PLAF7] | 11 |  |  |  | 3 | 3 | 3 | 2 | 383 | 45.5 | 9.70 |
| Q8IDG9 | PF3D7_1353100 | Uncharacterized protein OS=Plasmodium falciparum (isolate 3D7) OX=36329 GN=PF3D7_1353100 PE=4 SV=1 - [Q8IDG9_PLAF7] | 10 | 2 | 3 | 5 |  |  |  |  | 285 | 32.8 | 9.19 |
| Q8IJ28 | PF3D7_1038000 | Antigen UB05 OS=Plasmodium falciparum (isolate 3D7) OX=36329 GN=PF3D7_1038000.1 PE=4 SV=2 - [Q8IJ28_PLAF7] | 9 |  |  | 4 |  |  | 2 | 3 | 119 | 13.7 | 9.42 |
| Q8IBA0 | PF3D7_0826700 | Receptor for activated c kinase OS=Plasmodium falciparum (isolate 3D7) OX=36329 GN=PF3D7_0826700 PE=4 SV=1 - [Q8IBA0_PLAF7] | 9 |  |  |  | 2 | 5 | 2 |  | 323 | 35.7 | 6.71 |
| Q7KQL3 | PF3D7_1020900 | ADP-ribosylation factor 1 OS=Plasmodium falciparum (isolate 3D7) OX=36329 GN=ARF1 PE=1 SV=1 - [ARF1_PLAF7] | 8 |  |  |  | 2 | 3 | 2 |  | 181 | 20.9 | 6.21 |
| Q8IC01 | PF3D7_0708800 | Heat shock protein 110 OS=Plasmodium falciparum (isolate 3D7) OX=36329 GN=PF3D7_0708800 PE=4 SV=1 - [Q8IC01_PLAF7] | 7 |  |  |  |  | 3 | 1 | 2 | 873 | 99.9 | 5.69 |
| Q8I2X4 | PF3D7_0917900 | Heat shock protein 70-2 OS=Plasmodium falciparum (isolate 3D7) OX=36329 GN=PF3D7_0917900 PE=3 SV=1 - [Q8I2X4_PLAF7] | 7 | 2 | 3 | 2 |  |  |  |  | 652 | 72.3 | 5.31 |
| Q8IIJ8 | PF3D7_1116800 | Heat shock protein 101 OS=Plasmodium falciparum (isolate 3D7) OX=36329 GN=PF3D7_1116800 PE=1 SV=1 - [Q8IIJ8_PLAF7] | 7 | 2 |  | 3 |  | 2 |  |  | 906 | 102.8 | 9.14 |
| Q8IK15 | PF3D7_1002100 | EMP1-trafficking protein OS=Plasmodium falciparum (isolate 3D7) OX=36329 GN=PF3D7_1002100 PE=4 SV=1 - [Q8IK15_PLAF7] | 7 |  |  |  |  | 3 | 2 | 2 | 631 | 69.8 | 6.07 |
| P50498 | PF3D7_0206800 | Merozoite surface antigen 2 OS=Plasmodium falciparum (isolate 3D7) OX=36329 GN=MSA2 PE=1 SV=2 - [MSA2_PLAF7] | 6 |  | 2 | 2 |  |  |  | 2 | 272 | 27.9 | 5.58 |
| Q8IJ52 | PF3D7_1035700 | Duffy binding-like merozoite surface protein OS=Plasmodium falciparum (isolate 3D7) OX=36329 GN=PF3D7_1035700 PE=4 SV=1 - [Q8IJ52_PLAF7] | 6 |  |  |  | 3 | 2 |  | 1 | 697 | 80.2 | 5.10 |
| Q8IM16 | PF3D7_1407800 | Plasmepsin IV OS=Plasmodium falciparum (isolate 3D7) OX=36329 GN=PF3D7_1407800 PE=1 SV=1 - [Q8IM16_PLAF7] | 6 |  |  |  |  | 3 | 1 | 2 | 449 | 51.0 | 5.54 |

**Table S2. List of aggregation-prone *P. falciparum* proteins identified in extracellular vesicles from the 3D7 strain.** Aggregation propensity scores for EV-associated proteins were calculated using the TANGO algorithm under standard conditions (pH 7.4, 37 °C). Scores represent the intrinsic tendency of each protein to form β-aggregation-prone structures. Both high- and low-scoring proteins were retained to provide a comprehensive overview of EV-associated cargo. Low aggregation scores (<0.05) are included as reference proteins and to enable comparative interpretation across functional and biological categories.

| *Accession (UNIPROT)* | *Accession (PlasmoDB)* | *Protein name* | *TANGO score (AGG)* |
| --- | --- | --- | --- |
| Q76NM3 | PF3D7_1324900 | L-lactate dehydrogenase | 0.048 |
| Q7K6A4 | PF3D7_0922200 | S-adenosylmethionine synthase (EC 2.5.1.6) | 0.011 |
| C0H4V6 | PF3D7_0818200 | 14-3-3 protein | 0.020 |
| Q8I4X0 | PF3D7_1246200 | Actin-1 (Actin I) | 0.025 |
| Q8IKK7 | PF3D7_1462800 | Glyceraldehyde-3-phosphate dehydrogenase (EC 1.2.1.12) | 0.012 |
| Q9TY99 | PF3D7_0202000 | Knob-associated histidine-rich protein | 0.018 |
| Q8IIV2 | PF3D7_1105000 | Histone H4 | 0.009 |
| Q8I0P6 | PF3D7_1357000 | Elongation factor 1-alpha | 0.054 |
| Q8I492 | PF3D7_0500800 | Mature parasite-infected erythrocyte surface antigen | 0.041 |
| Q8IKF0 | PF3D7_1468700 | Eukaryotic initiation factor 4A (EC 3.6.4.12) | 0.040 |
| Q8IB24 | PF3D7_0818900 | Heat shock protein 70 | 0.022 |
| C6KSV0 | PF3D7_0610400 | Histone H3 | 0.002 |
| C6KT18 | PF3D7_0617800 | Histone H2A | 0.012 |
| Q8IIV1 | PF3D7_1105100 | Histone H2B | 0.007 |
| Q8IAX8 | PF3D7_0814200 | DNA/RNA-binding protein Alba 1 | 0.002 |
| Q8I6U8 | PF3D7_1016300 | Glycophorin-binding protein (GBP-130) | 0.039 |
| C0H571 | PF3D7_0929400 | High molecular weight rhoptry protein 2 | 0.182 |
| Q8I467 | PF3D7_0503400 | Cofilin/actin-depolymerizing factor homolog 1 | 0.034 |
| Q8IDQ9 | PF3D7_1343000 | Phosphoethanolamine N-methyltransferase (EC 2.1.1.103) | 0.012 |
| Q8IKW5 | PF3D7_1451100 | Elongation factor 2 | 0.032 |
| Q7KQK6 | PF3D7_1117700 | GTP-binding nuclear protein | 0.009 |
| C6KT34 | PF3D7_0619400 | Cell division cycle protein 48 homologue, putative | 0.047 |
| Q7KQL5 | PF3D7_1008700 | Tubulin beta chain (Beta-tubulin) | 0.012 |
| Q8IC05 | PF3D7_0708400 | Heat shock protein 90 | 0.048 |
| Q6LFH8 | PF3D7_0608800 | Ornithine aminotransferase (EC 2.6.1.13) (Ornithine-oxo-acid aminotransferase) | 0.057 |
| C6KTA4 | PF3D7_0626800 | Pyruvate kinase (EC 2.7.1.40) | 0.027 |
| Q8IDG8 | PF3D7_1353200 | Membrane-associated histidine-rich protein 2 | 0.028 |
| Q8I2G1 | PF3D7_0935900 | Ring-exported protein 1 | 0.020 |
| Q8I2F2 | PF3D7_0936800 | PRESAN domain-containing protein | 0.040 |
| A0A5K1K8J3 | PF3D7_1361800 | Glideosome-associated connector | 0.020 |

**Data S1. Raw data of Figures 2, 4 and 8.**
